# Construction of anti-HER2 affibody-directed CAR-NK and its synergistic effects with doxorubicin-loaded nanodrug against HER2-positive breast cancer

**DOI:** 10.3389/fimmu.2025.1692107

**Published:** 2026-01-12

**Authors:** Xuesong He, Zhaoyuan Liang, Qing Liu, Xiaofei Zhang, Hao Liang, Runqing Jia, Wang Sheng

**Affiliations:** 1 Beijing International Science and Technology, Cooperation Base of Antivirus Drug, College of Chemistry and Life Science, Beijing University of Technology, Beijing, China; 2 Department of Hepatobiliary Surgery, The Second Affiliated Hospital of Jiaxing University, Jiaxing, Zhejiang, China

**Keywords:** CAR-NK, NK92MI, affibody, DOX, nanoparticles

## Abstract

**Introduction:**

Chimeric antigen receptor (CAR)-engineered T or natural killer (NK) cells are a promising approach for cancer immunotherapy. The leading region of the CAR structure is generally a single-chain antibody (scFv) fragment specific for a tumor cell surface molecule, and other structures are rarely reported.

**Methods:**

In this study, we developed a novel anti-human epidermal growth factor receptor 2 (HER2) CAR-NK cell using an affibody molecule as the extracellular targeting domain instead of a conventional scFv. Affibody-based CAR-NK cells were generated from the NK-92 cell line. To enhance safety, CAR-NK cells were subjected to γ-irradiation, and their antitumor activity was further evaluated in combination with doxorubicin (DOX)-loaded nanoparticles.

**Results:**

Affibody-based CAR-NK cells exhibited effective cytotoxicity against HER2-positive breast cancer cells, comparable to that of anti-HER2 scFv-based CAR-NK cells. γ-Irradiation at 10 Gy effectively inhibited malignant proliferation of CAR-NK cells but significantly reduced their cytotoxic activity. Notably, incorporation of DOX-loaded nanoparticles markedly enhanced the killing capacity of irradiated CAR-NK cells, restoring and even amplifying their antitumor efficacy.

**Discussion:**

These findings demonstrate that affibody-based CAR-NK cells are a viable alternative to conventional scFv-based CAR constructs. Moreover, the combination of CAR-NK immunotherapy with chemotherapeutic nanomedicine effectively compensates for irradiation-induced cytotoxicity attenuation, offering a promising synergistic strategy for the treatment of HER2-positive breast cancer.

## 1 Introduction

Adoptive cellular therapy (ACT) has emerged as a powerful approach for controlling tumor growth, which uses immune cells to cure cancers (1, 2). As a branch of ACT, chimeric antigen receptor T (CAR-T) cell therapy has achieved good therapeutic effects in hematologic malignancies, particularly in B-cell lymphoma (3, 4). In 2017, the FDA approved two CAR-T cell therapies targeting CD19 for hematologic malignancies (5). Although CAR-T cells hold great promise, they are ineffective in the treatment of solid tumors, due to the role of tumor immune microenvironment, which poses significant barriers such as immunosuppressive signaling, physical stromal obstacles, and poor T cell infiltration (6, 7). In addition, CAR-T cell therapy has some shortcomings. The main problem is that the initial T cells must come from patients’ somatic cells to avoid graft versus host disease (GvHD) (8).

Natural killer (NK) cells are a subset of lymphocytes in the innate immune system that possess potent cytotoxic function, independent of major histocompatibility complex (MHC) restriction. Beyond that, NK cells are also suitable for the modification of chimeric antigen receptors (CARs), making them a promising platform for adoptive immunotherapy. Compared to T lymphocytes, NK cells have a lower risk of inducing GvHD, thereby offering advantages for allogeneic, off-the-shelf CAR-based therapies (9–11). At present, NK cells can be sourced from peripheral blood, pluripotent stem cells, or established NK cell lines (12). Among them, NK-92 is a cell line with stable genetic characteristics and can proliferate infinitely, which can solve the shortcomings of insufficient number of NK cells in patients. NK-92, first established in 1992, is an interleukin-2 (IL-2)-dependent immortal cell line derived from a patient with non-Hodgkin’s lymphoma (13). Compared with primary NK cells, NK-92 cells are homogeneous, can be easily expanded, and are more amenable to genetic modification. NK-92 cells also exhibit strong cytotoxicity against a broad range of tumor cells and express low levels of inhibitory receptors, enabling improved tumor cell killing (10). In the context of CAR-NK cell therapy, NK cell lines have been widely explored, and CAR-NK cells based on the NK-92 platform have already advanced into clinical trials (13–15).

However, the requirement for IL-2 supplementation in culture remains a limitation. To address this, researchers have developed an IL-2-autonomous variant, NK92-MI, by introducing the IL-2 gene into NK-92 cells, allowing for continuous proliferation without external IL-2 support (16). These properties make NK-92 and its derivatives valuable tools for both preclinical research and clinical immunotherapy applications.

The traditional CAR structure comprises an extracellular antigen recognition domain, a spacer domain, a transmembrane region, and an intracellular signaling domain. The extracellular antigen recognition domain is typically a single-chain variable fragment (scFv), which is derived from the heavy and light chain variable regions (VH and VL) of antibodies (17). Similar to antibodies, scFv can target and recognize tumor antigens, which is the most common extracellular structure of CAR (17, 18). In addition to the scFv structure, alternative scaffolds such as adnectins, affibodies, anticalins, ankyrin repeats, and nanobodies have been explored. These alternative structures exhibit superior or comparable affinity and specificity, offering promising alternatives for CAR-based therapies (19–24).

Affibodies are a class of small-molecule affinity proteins with triple helix domains derived from staphylococcal protein A. It was first proposed as early as 20 years ago. To date, more than 40 affibodies have been developed for use in different fields such as experimental diagnosis, molecular imaging, and targeted therapy etc (22). The first to be used in clinical imaging is the HER2-specific affinity Z_Her2:342_, which can show HER-2 overexpressing tumor metastatic lesions (25). Given their high affinity and small size, affibodies have also been integrated into nanoparticle-based drug delivery systems to enhance therapeutic efficacy, particularly in HER2-positive breast cancer (26). In 2010, Joachim Feldwisch et al. optimized on the basis of the Z_Her2:342_ structure and designed an affibody structure ABY-025 with enhanced binding properties (27). Despite the promising potential of affibodies in targeting HER2-positive tumors, their use in adoptive immunotherapy, particularly in CAR-NK cell-based treatments, has not been explored.

In this study, we developed a novel HER2-specific CAR-NK construct using the high-affinity affibody ABY-025 as the extracellular antigen recognition domain. Compared with the unoptimized Z_HER2:342_ affibody, ABY-025 exhibits improved stability and binding affinity. To assess its functional capacity, we directly compared affibody-based CAR-NK cells with conventional scFv-based CAR-NK cells. Our findings demonstrate that the affibody-based CAR-NK cells exhibit comparable cytotoxic activity to their scFv counterparts, indicating that affibodies can serve as effective and functional targeting moieties in CAR constructs without impairing NK cell killing efficiency.

**Scheme 1:**
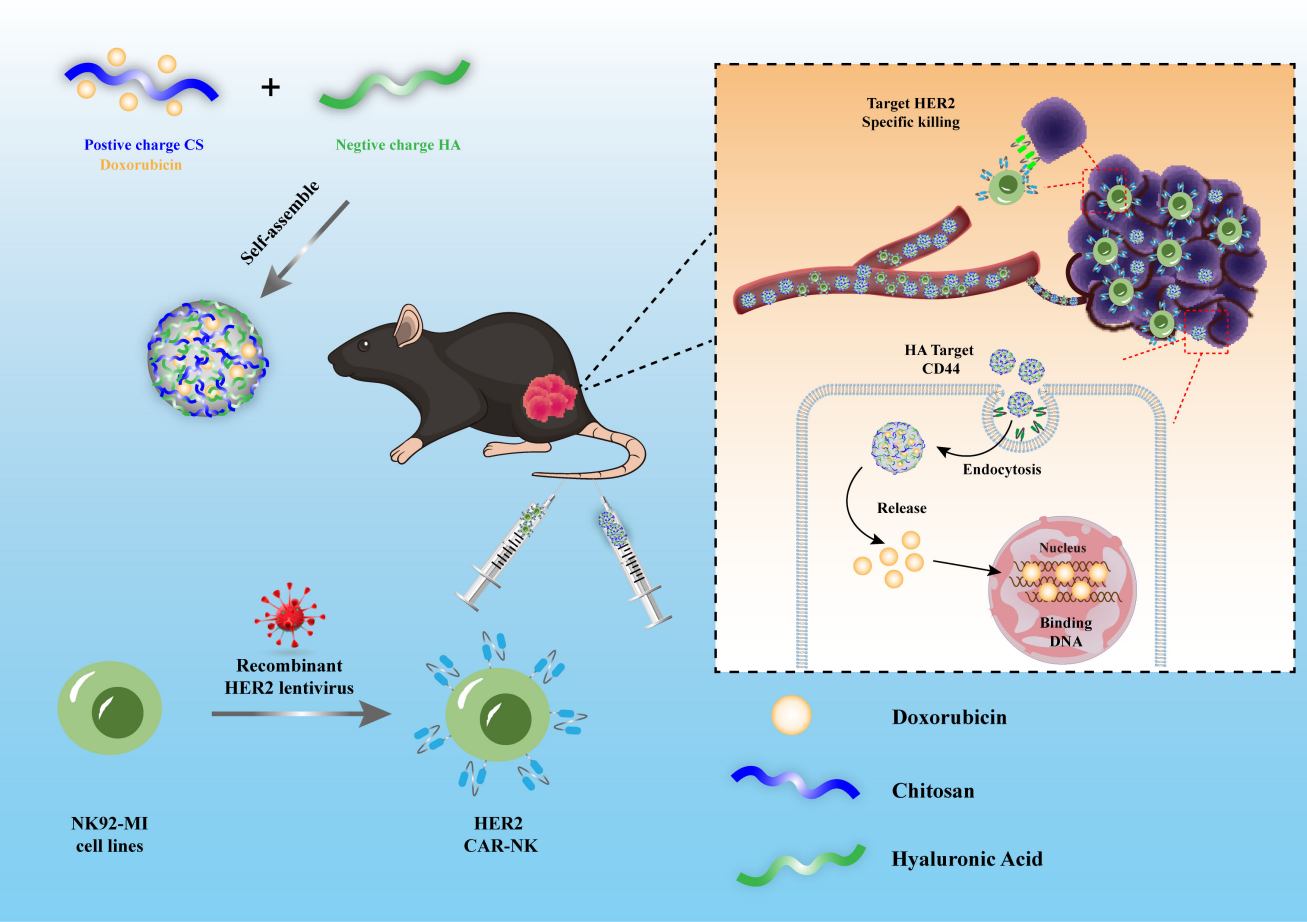
Synergistic antitumor effect of HER2-targeted CAR-NK cells and hyaluronic acid (HA)- chitosan (CS)-based doxorubicin-loaded nanoparticles for the treatment of breast cancer. Recombinant HER2 lentivirus is used to transduce NK92-MI cells, generating HER2-specific CAR-NK cells. Positively charged CS and negatively charged HA self-assemble into nanoparticles loaded with the chemotherapeutic drug doxorubicin. This dual therapeutic system is administered intravenously into breast tumor-bearing mice. After injection, both HER2 CAR-NK cells and HA-CS nanoparticles circulate through the bloodstream and accumulate at the tumor site. The nanoparticles target tumor cells via HA–CD44 interaction and undergo endocytosis, releasing doxorubicin intracellularly to induce tumor cell cytotoxicity. Meanwhile, HER2 CAR-NK cells specifically recognize and kill HER2-positive tumor cells. The combination therapy exerts a synergistic antitumor effect through both immune-mediated and chemotherapeutic mechanisms.

Given the need for γ-irradiation of NK-92MI cells to prevent uncontrolled proliferation prior to clinical use, a significant reduction in cytotoxicity was observed post-irradiation. We proposed a combination therapy that integrates affibody-based CAR-NK with nanoparticle-mediated chemotherapy to address this therapeutic limitation. Doxorubicin (DOX), a standard chemotherapeutic agent for breast cancer, suffers from dose-limiting systemic toxicity. Nanoparticle formulations of DOX have been developed to improve tumor selectivity and reduce adverse effects (28). Based on our previous work in DOX nanomedicine (29), we co-administered DOX-loaded nanoparticles with irradiated CAR-NK cells to evaluate potential synergistic effects (**Scheme 1**). Our results reveal that this combination therapy significantly restores and enhances the antitumor efficacy of irradiated CAR-NK cells against HER2-positive breast cancer cells, while maintaining favorable safety.

In summary, this study proposes a novel combinatorial therapeutic strategy that integrates affibody-based CAR-NK cells with doxorubicin-loaded nanoparticles. By leveraging the high-affinity targeting capability of the ABY-025 affibody and the enhanced delivery profile of nanoparticle-formulated DOX, this platform demonstrates potent and synergistic antitumor activity against HER2-positive solid tumors, improving the efficacy of adoptive immunotherapy in solid malignancies.

## 2 Materials and methods

### 2.1 Cell lines and culture media

Human MCF-7, B-T474, and SK-BR-3 breast carcinoma cells were all purchased from the National Infrastructure of Cell Line Resource (Beijing, China). Human natural killer cell NK-92MI was purchased from ATCC (Manassas, VA, USA). Lenti-X 293T cells were purchased from Clontech (Palo Alto, CA, USA). MCF-7 cells and Lenti-X 293T cells were cultured in DMEM (Gibco, Grand Island, NY, USA). SK-BR-3 cells and BT474 cells were cultured in RPMI 1640 (Gibco, Grand Island, NY, USA). All media of the above were supplemented with 10% FBS (Gibco, USA), 100U/ml penicillin, and 100 μg/ml streptomycin (Gibco, USA). NK92-MI cells were cultured in α-MEM supplemented with 0.2 mM inositol (Sigma, Germany), 0.1 mM 2-mercaptoethanol (Sigma, Germany), 0.02 mM folic acid (Sigma, Germany), 12.5% FBS, and 12.5% horse serum (Gibco, USA).

### 2.2 Generation of CAR-expressing NK-92-MI cells

The HER2-targeted scFv-based CAR was constructed based on the previously reported design by Professor Liu (30). The scFv sequence was codon-optimized and modified at the 5` ends with a signal peptide and a Kozak sequence to ensure efficient translation. A C-terminal avian myelocytomatosis viral oncogene (Myc) tag was fused to the scFv, followed by the human CD8α hinge domain, CD28 transmembrane region, and the CD3ζ intracellular signaling domain. Similarly, two affibody-based CAR constructs (designated as Affibody 1 CAR and Affibody 2 CAR) were designed according to the reported sequences by Joachim and Charles (27, 31), in which the scFv was replaced with HER2-specific affibody domains. The codon-optimized CAR gene fragments were synthesized by Sangon Biotech (Shanghai, China) and cloned into the lentiviral vector pLVX-Puro (Clontech, Palo Alto, CA, USA). Lentiviral particles were produced in Lenti-X 293T cells using the Lipofectamine™ 3000 transfection system (Thermo Fisher Scientific, USA), according to the manufacturer’s instructions with modifications. One day prior to transfection, Lenti-X 293T cells were seeded into 10 cm culture dishes at a density of 1 × 10^7^ cells in complete DMEM medium supplemented with 10% FBS and incubated overnight. On the day of transfection, the culture medium was replaced with 5 mL of fresh complete DMEM without antibiotics. For each 10 cm dish, a DNA-P3000 mixture was prepared by adding 6 μg of lentiviral transfer plasmid (CAR-pLVX), 4.5 μg of packaging plasmid psPAX2, and 1.5 μg of envelope plasmid pMD2.G into 1.5 mL of Opti-MEM (Gibco, USA) containing 24 μL of P3000 reagent. In parallel, 40 μL of Lipofectamine 3000 reagent was diluted in 1.5 mL of Opti-MEM. The DNA-P3000 solution was slowly added to the Lipofectamine 3000 solution, gently mixed by inversion, and incubated for 15 min at room temperature to allow the formation of DNA-lipid complexes. The transfection complexes were then added dropwise to the Lenti-X 293T cells, and incubated at 37°C with 5% CO_2_. After 6 h of incubation, the medium was replaced with 10 mL of fresh complete DMEM. Lentiviral supernatants were harvested at 48 and 72 h post-transfection, centrifuged at 5,000g for 10 min at 4°C to remove cell debris, and filtered through a 0.45μm PVDF filter. The viral supernatant was then concentrated by ultracentrifugation at 100,000g for 90 min at 4°C. The viral pellet was resuspended in 200 μL of complete medium and stored at −80°C.

To determine the functional viral titer, a puromycin-resistant colony formation assay was performed on Lenti-X 293T cells using serial 10-fold dilutions (10^-2^–10^-8^) of the concentrated lentivirus in the presence of polybrene (6 μg/mL). After overnight infection and puromycin (1 μg/mL) selection for 3 days, crystal violet staining was used to quantify colony numbers, and titers were calculated in transducing units per milliliter (TU/mL). Viral titers formula is TU/mL= number of colonies × dilution factor/infection volume (mL). Representative titers were 2.3×10^8^, 4.4×10^8^, and 3.8×10^8^ TU/mL for scFv CAR, Affi1 CAR, and Affi2 CAR, respectively. NK-92MI cells were transduced at a multiplicity of infection (MOI) of approximately 20. Briefly, cells were seeded at 2×10^5^ cells/mL in a 6-well plate, and 200 μL of concentrated lentivirus was added per well together with polybrene (6 μg/mL). Cells were incubated for 24 h at 37°C and 5% CO_2_, followed by gentle centrifugation (1000 rpm, 5 min) and replacement with fresh complete α-MEM medium containing 12.5% fetal bovine serum and 12.5% horse serum. 72 h after transduction, stable CAR-expressing NK-92MI cells were selected using puromycin at 1.5 μg/mL for 3 days until non-transduced cells were eliminated. Surviving cells were expanded and maintained for downstream experiments.

**Figure 1:**
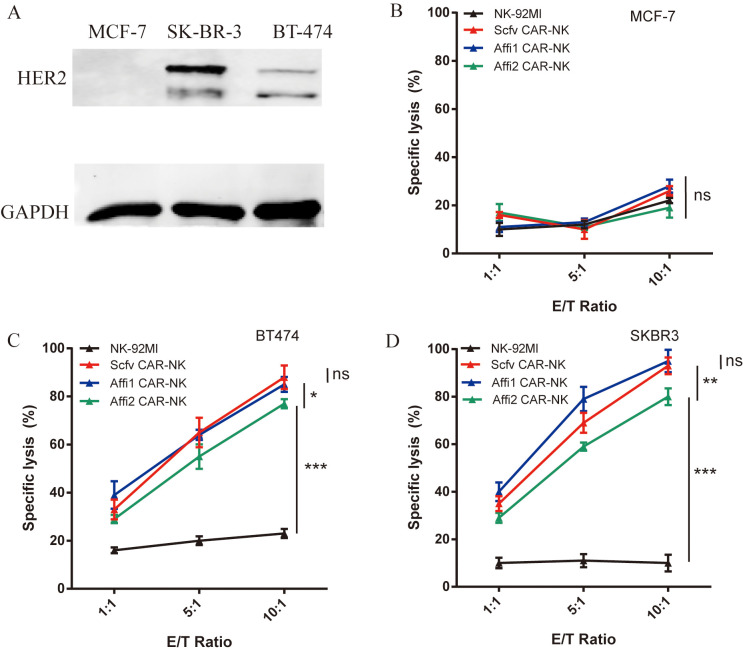
The expression of HER-2 in different breast cancer cell lines and the killing effect of three kinds of CAR-NK on various breast cancer cells. **(A)** Using western blot to detect the expression of HER-2 on the surface of three breast cancer cells, the antibody used anti-HER-2 and anti-GAPDH monoclonal antibodies. **(B–D)** Cell lysis efficiency of effector cell (NK-92MI, scFv CAR-NK, Affi1 CAR-NK, Affi2 CAR-NK) and target cell (MCF-7, BT-474, SK-BR-3) incubate for 6 hours at E:T 1:1, 5:1 and 10:1. Data are presented as the mean ± SD, and significant differences between groups were measured by using two-way analysis of variance (ANOVA). * P<0.05, **P <0.01, ***P < 0.001, ns not significant, n=3.

CAR expression in NK-92MI cells was assessed by flow cytometry using a FITC-conjugated anti-Myc tag antibody (Invitrogen, CA, USA) on a BD Calibur flow cytometer (BD Biosciences, San Jose, CA, USA). For flow cytometric analysis, debris was excluded by forward and side scatter (FSC-A/SSC-A) gating, and single cells were identified using FSC-A versus FSC-H to eliminate doublets. Unstained NK-92MI cells and FITC isotype controls were used to establish the negative threshold, and CAR-positive cells were defined as events exceeding the 99th percentile of the isotype control. CAR expression levels were quantified as both the percentage of CAR-positive cells and mean fluorescence intensity (MFI), and these quantitative data are presented in **Figure 1**, **Supplementary Figure S1**. All experiments were performed using three independent biological replicates (n = 3), each analyzed in technical duplicates.

### 2.3 *In vitro* cytotoxicity assays

The HER-2 protein expression level of SK-BR-3, BT-474, and MCF-7 cells was tested by western blot using anti-human HER2 antibody (Abcam, MA, USA). The cytotoxicity of three kinds of CAR-NK cells towards HER-2 positive target tumor cells was analyzed using a lactate dehydrogenase (LDH) assay (Dojindo, Kumamoto, Japan). The ratios of effect cells and target cells were 1:1, 5:1, and 10:1. Spontaneous LDH release (effector-only and target-only wells) and maximum LDH release (target cells treated with lysis buffer) were included as controls. Absorbance at 490 nm was measured using a microplate reader (Perkin Elmer, USA). Specific lysis was calculated with the following formula: [(experimental LDH release–effector cells spontaneous LDH release–target cells spontaneous LDH release)/(target cells maximum LDH release–target cells spontaneous LDH release)] ×100%.

### 2.4 Time-lapse imaging

HER-2 positive SKBR-3 were used as target cells and Affi 1 CAR-NK were used as effector cells. 1×10^5^ target cells were seeded in a 35 mm confocal dish 24 hours before adding effector cells. Time-lapse imaging was carried out using a Cell Observer system (Carl Zeiss, Göttingen, Germany) with temperature and gas control. Phase-contrast images of each position were taken every 2 minutes for 4 hours with a 10× Neofluar objective (Carl Zeiss) and an Axio CamHRm camera with Carl Zeiss Axio Vision 4.8 software.

### 2.5 Irradiation of NK-92 cells, cytotoxicity assays

#### 2.5.1 Irradiation of CAR-NK cells

NK92-MI, scFv-, Affi1- and Affi2-CAR-NK cells in logarithmic growth phase were collected, washed, and resuspended in α-MEM complete medium. Cells were plated in 100 mm culture dishes. Cells were irradiated at room temperature using a medical linear accelerator (Siemens Primus-M, Germany) delivering 6 MV X-rays at a dose rate of 2 Gy/min. Radiation doses of 0, 2, 5, and 10 Gy were applied. Immediately after irradiation, cells were washed, resuspended in fresh α-MEM complete medium, and cultured in a humidified incubator at 37°C with 5% CO_2_.

#### 2.5.2 Cell proliferation assessment

To determine the effect of irradiation on cell growth, irradiated cells were collected immediately after treatment, stained with trypan blue, and adjusted to 4×10^5^ cells/mL. Cells were seeded into 24-well plates and viable cells were counted at 0, 24, 48, 72, and 96 h post-irradiation using a hemocytometer. Only trypan blue-negative cells were recorded as live cells.

#### 2.5.3 Cell viability assay (CCK-8)

Cell viability was assessed using a CCK-8 assay (Dojindo, Japan). Cells (1 × 10^3^ cells/well) were seeded into 96-well plates immediately after irradiation. At each time point (0, 24, 48, 72, and 96 h), 10 μL of CCK-8 reagent was added to each well and incubated at 37°C for 1 h. Absorbance was measured at 450 nm using a microplate reader (Perkin Elmer, USA).

#### 2.5.4 Cytotoxicity assay after irradiation

The cytotoxic activity of irradiated NK-92MI and CAR-NK cells against breast cancer cells was determined using an LDH release assay (Dojindo, Japan). Effector cells were harvested 24 h post-irradiation and co-cultured with target cells at effector-to-target (E:T) ratios of 1:1, 5:1, and 10:1 in 96-well plates for 6 h at 37°C with 5% CO_2_. Spontaneous LDH release (effector-only and target-only wells) and maximum LDH release (target cells treated with lysis buffer) were included as controls. After incubation, 50 μL of supernatant was transferred to a new plate and mixed with 50 μL of LDH reaction solution. Absorbance at 490 nm was recorded, and cytotoxicity was calculated using the formula provided in Section 2.3.

#### 2.5.5 Cytokine secretion after irradiation

To evaluate irradiation effects on immune function, irradiated and control NK cell supernatants were collected 24 h post-treatment and centrifuged to remove debris. The concentrations of granzyme B, IFN-γ, perforin, and TNF-α were quantified using human ELISA kits (Abcam, Cambridge, MA, USA) according to the manufacturer’s instructions. Standard curves were generated for each cytokine, and absorbance at 450 nm was measured using a microplate reader (Perkin Elmer, USA).

### 2.6 Encapsulation of doxorubicin by nanoparticles and cytotoxicity assays

Doxorubicin nanoparticles (DNP) were synthesized, and characterized as described previously (29). The synergistic therapeutic effect of nanomedicine and CAR-NK was tested using a lactate dehydrogenase (LDH) assay (Dojindo, Kumamoto, Japan). SK-BR-3 and MCF-7 were target cells, and Affi1 CAR-NK were effector cells. NK92-MI was used as a control. DNPs were added to the medium 24h earlier than NK cells. The concentration of DNPs was 0.1 μg/mL. The E: T ratios were 1:1, 5:1, and 10:1. The Specific lysis calculation method was the same as above.

### 2.7 *In vivo* anti-HER-2 positive tumor experiments

Four to six-week-old, female NOD/SCID (NSG) mice were purchased from Vital River Laboratory Animal Technology Co., Ltd. (Beijing, China). All the mice were injected subcutaneously on their right flank with 1×10^7^ SK-BR-3 cells mixed with the same volume of Matrigel (BD Bioscience, San Jose, USA). When the tumor volume reached approximately 100 mm^3^, mice were randomly assigned into eight groups (n = 5 per group) using a computer-generated randomization schedule. All treatments and tumor measurements were performed in a blinded manner; the investigators administering treatments and measuring tumor size were unaware of group allocation. DNP, DNP+NK (I) (I represents 10 Gy dose irradiation treatment), and DNP+CAR-NK (I) groups of mice were injected with 100μL DNP (1 mg/kg) intravenously, and the control group was injected with 100μL PBS on day 0. On Day 1, 1×10^7^normal NK, normal CAR-NK, and irradiated CAR-NK cells were injected intravenously into different groups. 1×10^7^ different groups of cells were injected intravenously for the second and third treatment on day 6 and day 10. Tumor growth was measured every week (tumor volume = length×width^2^×0.5), and animals were sacrificed when the tumor reached 1 cm in diameter.

### 2.8 Biological safety evaluation

After mice were sacrificed, residual CD45^+^CD56^+^ NK cells in blood were tested by flow cytometry with a BD Calibur Flow Cytometer (BD Biosciences, San Jose, CA). For the *in vivo* toxicity assay, organs (heart, lung, liver, kidney, spleen) were collected for hematoxylin and eosin staining.

### 2.9 Statistical analyses

Statistical analyses were performed in GraphPad Prism 8. Data are presented as means ± SD or means ± SEM as the figure legends state. One-way or two-way analysis of variance (ANOVA) was used to examine the statistical difference between the groups, unless otherwise stated. Statistical significance is indicated as follows: *, P < 0.05; **, P < 0.01; ***, P < 0.001; ****, P < 0.0001; ns, not significant.

## 3 Results

### 3.1 Construction of HER-2 specific CAR-NK cell lines

**Figure 2:**
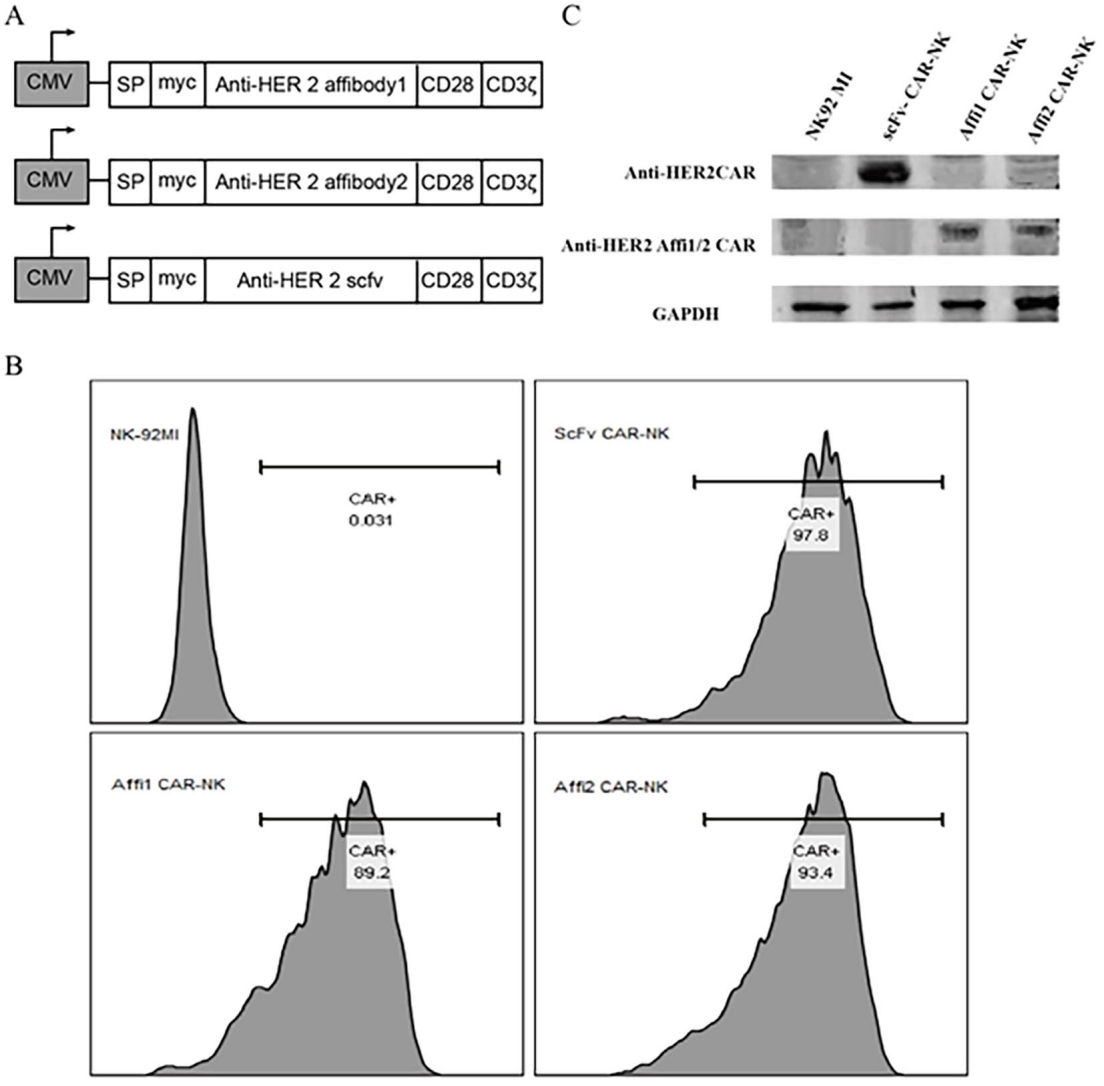
The structure and expression of CAR in NK92-MI cells. **(A)** Schematic diagram of three CAR structures using Affibody 1, Affibody 2, and scFv as the extracellular domain. **(B)** CAR expression efficiency of NK92-MI cells was detected by flow cytometry. Anti-myc antibody was labeled with FITC. **(C)** The expression of three CARs was detected by western blot. Anti-myc tag monoclonal antibody and anti-GAPDH monoclonal antibody were used. Based on the DNA sequence, the size of the scFv CAR protein was estimated to be approximately 55 kDa, while Affi1 CAR and Affi2 CAR proteins were approximately 34 kDa. The size of the proteins in the first lane is about 55 kDa, and the size of the proteins in the second lane is about 34 kDa.

HER2-specific CAR-NK cells were established from NK92-MI cells via lentiviral transduction, utilizing a second-generation CAR construct encoding different HER2-targeting domains. The extracellular domain of the CAR included either the engineered affibody ABY-025 (Affi1), the unmodified HER2:342 affibody (Affi2), or a HER2-specific single-chain variable fragment (scFv). The CAR architecture consisted of a myc epitope tag, CD8α-derived hinge and transmembrane domains, the intracellular domain of human CD28, and the CD3ζ signaling motif (**Figure 2A**). After transduction and puromycin selection, CAR surface expression was verified by flow cytometry using FITC-conjugated anti-myc antibody, high positivity rates were observed in scFv CAR-NK, Affi1 CAR-NK, and Affi2 CAR-NK as 97.8%, 89.2% and 93.4%, respectively (**Figure 2B**, **Supplementary Figure S6**). Immunofluorescence imaging (**Supplementary Figure S1**) and western blotting (**Figure 2C**) further validated CAR protein expression. The estimated molecular weights were ∼55 kDa for scFv CAR, and ∼34 kDa for both Affi1 and Affi2 CARs, confirming proper construct integrity. These indicated the successful generation of three HER2-targeted CAR-NK cell lines.

### 3.2 Cytotoxicity of CAR-NK cells on different breast cancer cell lines

Western blot analysis revealed high HER2 expression in SK-BR-3 and BT-474 cells, but not in MCF-7 cells, which served as a HER2-negative cell control (**Figure 1A**). Dual bands were detected due to the dimerization of HER2. Among HER2^+^ lines, SK-BR-3 exhibited a higher HER2 expression than BT-474. The cytolytic activity of CAR-NK cells against HER2-positive breast cancer cells was assessed via LDH release assays at various effector-to-target (E: T) ratios (1:1, 5:1, and 10:1). Untransfected NK-92MI was used as a negative cell control. As shown in **Figure 1B**, low and similar cell cytolytic activity was observed in either scFv, Affi1, and Affi2 CAR-NK or NK-92MI cells, while incubated with HER2-negative MCF-7 cells, indicating that no cytolytic activity was observed in HER2-negative MCF-7 cells upon NK cell treatment. In contrast, dramatic cell cytolytic activity was obtained in both HER2^+^ BT-474 and SK-BR-3 cells (**Figures 1C, D**) when incubated with CAR-NK cells, and showed a dose-dependent manner. However, low cell cytolytic activity was observed while treated with untransfected NK-92MI cells, suggesting that scFv, Affi1, and Affi2 CAR-NK killed HER2^+^ breast cancer cells through HER2-mediated targeting. In addition, higher cytolytic phenomenon was observed in scFv and Affi1 CAR-NK treatment than that treated by Affi2 CAR-NK in either BT-474 or SK-BR-3 cells, suggesting higher HER2 recognition or binding ability of scFv and Affi1 CAR than Affi2 CAR. Otherwise, higher cytolytic rates were found in scFv and Affi1 CAR-NK treated SK-BR-3 cells than in BT-474 cells, which is consistent with higher expression of HER in SK-BR-3 cells than in BT-474 cells.

### 3.3 Live cell imaging to observe the killing process of Affi1 CAR-NK cells

**Figure 3:**
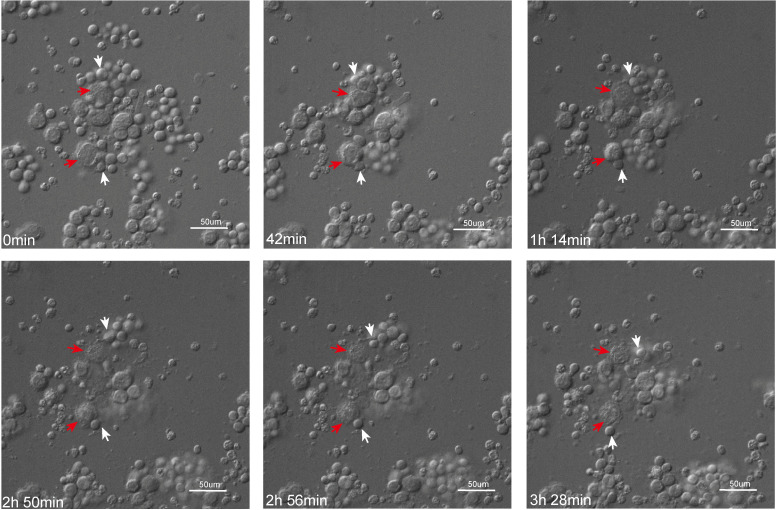
Time-lapse imaging of Affi1 CAR-NK–mediated killing of HER2-positive tumor cells. Affi1 CAR-NK cells (effector) and SK-BR-3 cells (target) were co-cultured at an E:T ratio of 1:1, and live-cell imaging was performed at the indicated time points (0 min, 42 min, 1 h 14 min, 2 h 50 min, 2 h 56 min, and 3 h 28 min). White arrows indicate Affi1 CAR-NK cells, and red arrows indicate SK-BR-3 tumor cells. Progressive reduction of target cells over time reflects Affi1 CAR-NK cytotoxic activity. Scale bar = 50 μm.

Live-cell imaging was adopted to dynamically monitor Affi1 CAR-NK-mediated cytotoxicity against SK-BR-3 cells. As shown in **Figure 3**, Affi1 CAR-NK cells formed stable contacts with target cells within 42 minutes, followed by morphological changes indicative of apoptosis and membrane rupture, culminating in target cell lysis. The effector cell detached and engaged a new target, demonstrating serial killing capability. These time-lapse observations confirm the HER2-specific recognition and cytotoxic action of Affi1 CAR-NK cells.

### 3.4 Optimization of irradiation dose and evaluation of CAR-NK cell cytotoxicity

**Figure 4:**
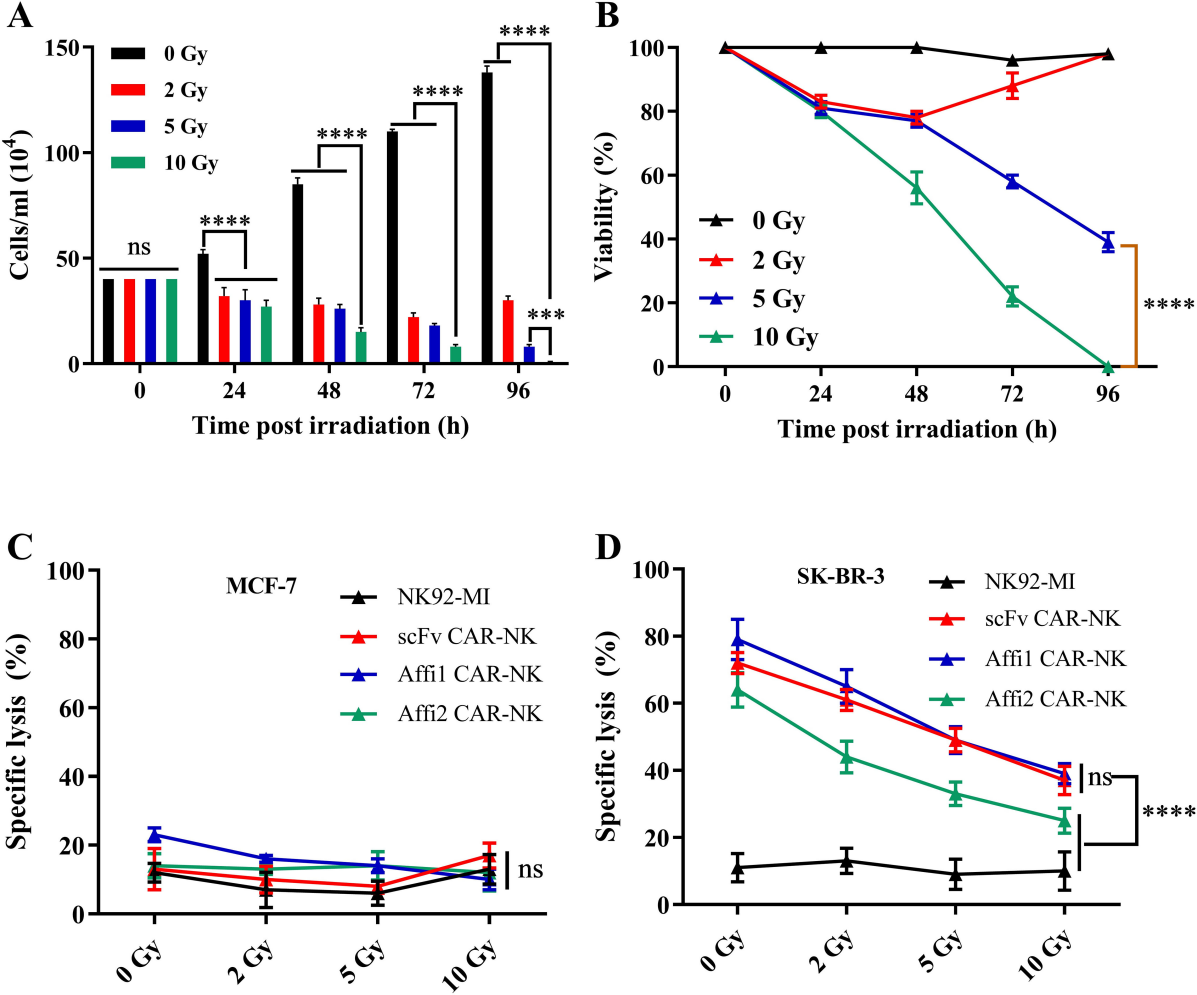
Effects of different doses of irradiation on the proliferation of Affibody 1 CAR-NK cells and the killing function of CAR-NK cells after irradiation. **(A)** Changes in the number of Affi1 CAR-NK cells after different doses of radiation (0, 2, 5, 10 Gy) during different times (0, 24, 48, 72, 96 hours) **(B)** CCK8 was used to measure the change of cell activity of Affi1 CAR-NK after irradiation **(C)** Killing of target cells MCF-7 by effector cells after irradiation with different doses (0, 2, 5, 10 Gy) **(D)** Killing of target cells SK-BR-3 by effector cells after different doses of irradiation (0, 2, 5, 10 Gy). Data are presented as the mean ± SD, and significant differences between groups were measured using two-way analysis of variance (ANOVA). ****P < 0.0001 ns not significant, n=3.

Since NK-92 is a continuously proliferating malignant cell line and NK-92MI shares this characteristic, clinical application requires pre-treatment to ensure biosafety. Currently, irradiation is commonly employed to eliminate the tumorigenic potential of NK-92 cells while preserving their cytotoxic function (13). To ensure biosafety, Affi1 CAR-NK cells were irradiated at 0, 2, 5, and 10 Gy. Cell counts and CCK-8 assays showed that 10 Gy completely halted proliferation, while 2 Gy and 5 Gy allowed partial recovery (**Figures 4A, B**). Thus, 10 Gy was selected for *in vivo* application. Functional assessment revealed that irradiation did not affect cytotoxicity against HER2-negative MCF-7 cells, but significantly impaired killing of HER2-positive SK-BR-3 cells (**Figures 4C, D**). These findings suggest that irradiation effectively eliminates proliferative potential and increases biosafety, which may eventually attenuate the effector function of NK cells and underscore the need for adjunct strategies to maintain therapeutic efficacy.

### 3.5 Effects of irradiation on cytokine secretion

**Figure 5:**
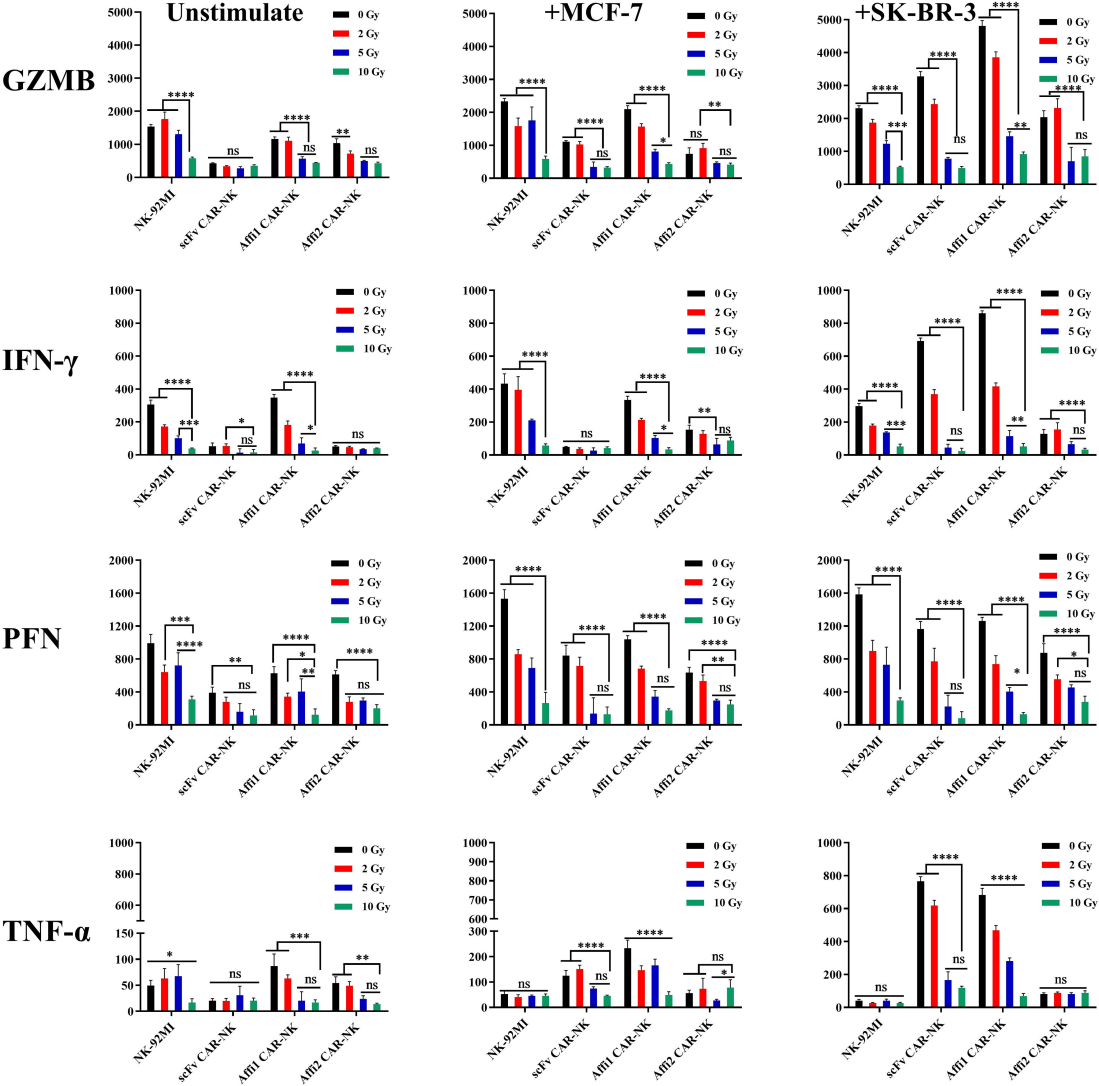
Effects of irradiation on cytokines secretion of NK-92 MI, scFv CAR-NK, Affi1 CAR-NK, Affi2 CAR-NK at different irradiation doses (0, 2, 5, 10 Gy) as effector cells to unstimulated cells or to MCF-7, SK-BR-3 as the target cells in a Petri dish for 6h The content of Granzyme B (GZMB), Interferon gamma (IFN-γ), Perforin (PRF), and Tumor necrosis factor alpha (TNF-α) in different groups of supernatants were detected by ELISA. Data are presented as the mean ± SD, and significant differences between groups were measured using two-way analysis of variance (ANOVA). * P<0.05, **P <0.01, ***P < 0.001, ****P < 0.0001 ns not significant, n=3.

We next assessed cytokine production in irradiated and non-irradiated CAR-NK cells after 6-hour co-incubation with MCF-7 or SK-BR-3 cells. The secretion of granzyme B, IFN-γ, TNF-α, and perforin was measured by ELISA. Untransfected NK92-MI cells were used as a killing cell control, and MCF-7 was used as an HER2-negative target cell control. As shown in **Figure 5**, decreased secretion of granzyme B, IFN-γ, TNF-α, and perforin was particularly observed in all irradiated cells, compared to non-irradiated cells, and showed a dose-dependent manner. The obtained results are especially correlated with the above cell viability analysis. Compared to unstimulated NK-92 MI and CAR-NK cells, no significant differences in the secretion of granzyme B, IFN-γ, TNF-α, and perforin were found upon stimulation by incubation with HER2-negative MCF-7 cells, suggesting that MCF-7 cells are not able to stimulate the secretion of such cytokines. In contrast, compared to these controls, remarkable increased secretion of granzyme B, IFN-γ, and TNF-α was observed in scFv and Affi1 CAR-NK cells, while stimulated by incubation with HER2-positive SK-BR-3 cells. The data suggested that the secretion of granzyme B, IFN-γ, and TNF-α is regulated by a designed HER2 CAR-mediated artificial signaling pathway. However, no obvious differences in the secretion of granzyme B, IFN-γ, and TNF-α in NK-92MI and affi2 CAR-NK cells upon incubation with SK-BR-3 cells, which is consistent with the absence of HER2 CAR in NK-92MI cells and low recognition ability of affi2 CAR to the HER2 molecule in affi2 CAR-NK cells. The results revealed that the secretion of granzyme B, IFN-γ, and TNF-α showed a HER2 CAR-dependent manner. Moreover, compared to the controls, no obvious differences were observed in the secretion of perforin in all NK cells while incubated with SK-BR-3 cells, indicating that the designed HER2 CAR has limited effects on the regulation of perforin expression and shows a CAR-independent release pattern. Collectively, these results indicate that irradiation dampens the immune activation and cytokine output of CAR-NK cells, potentially compromising therapeutic performance.

### 3.6 *In vitro* killing effects of doxorubicin nanoparticles combined with CAR-NK

**Figure 6:**
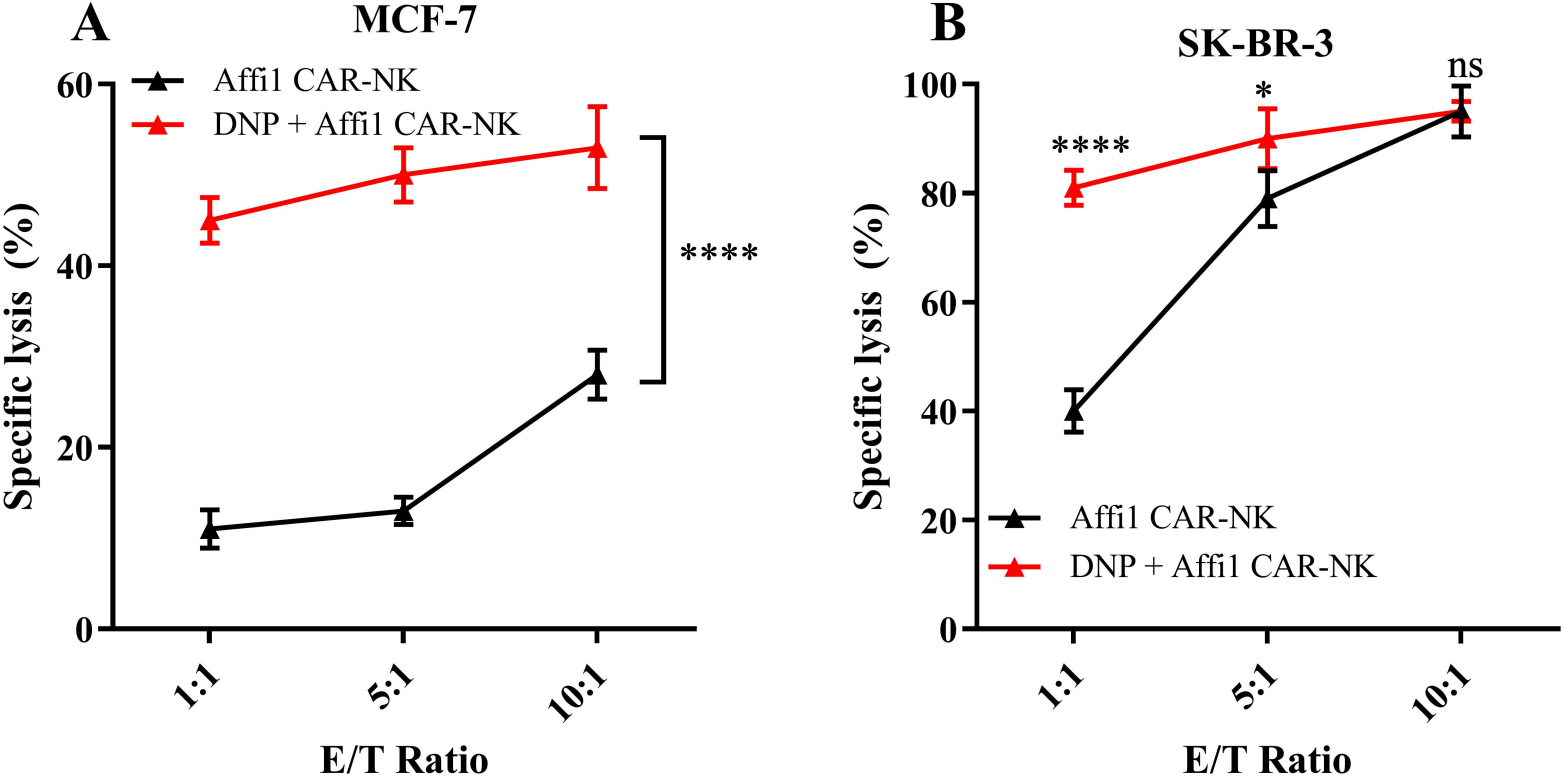
Affi1 CAR-NK cell killing after doxorubicin nano-drug treatment **(A)** MCF-7 was used as the target cell, and DNP was added in advance to incubate for 12h. After that, effector cells Affi1CAR-NK were added at different effector-to-target ratios (1:1, 5:1, 10:1), and the cytotoxicity was detected by LDH after 6h. **(B)** SK-BR-3 was used as the target cell, and DNP was added in advance to incubate for 12h. After that, effector cells Affi1CAR-NK were added at different effector-to-target ratios (1:1, 5:1, 10:1), and the cytotoxicity was detected by LDH after 6h. Data are presented as the mean ± SD, and significant differences between groups were measured using two-way analysis of variance (ANOVA). * P<0.05, ****P < 0.0001 ns not significant, n=3.

Previous work by Erik et al. demonstrated that tumor cells treated with doxorubicin exhibit increased susceptibility to NK cell-mediated cytotoxicity (32). We tested whether doxorubicin-loaded nanoparticles (DNP) could restore cytotoxic function. DNPs were formulated with hyaluronic acid-chitosan, validated for size and stability (**Supplementary Figures S2**, **S3**). LDH assays demonstrated that combination therapy significantly enhanced cytotoxicity against HER2-negative MCF-7 cells (**Figure 6A**), and additive effects were observed in HER2-positive SK-BR-3 cells at lower E: T ratios (**Figure 6B**). These findings support the use of DNPs to sensitize tumor cells to NK-mediated killing, particularly when CAR-NK potency is compromised.

### 3.7 *In Vivo* antitumor efficacy of combined DNP and CAR-NK therapy

**Figure 7:**
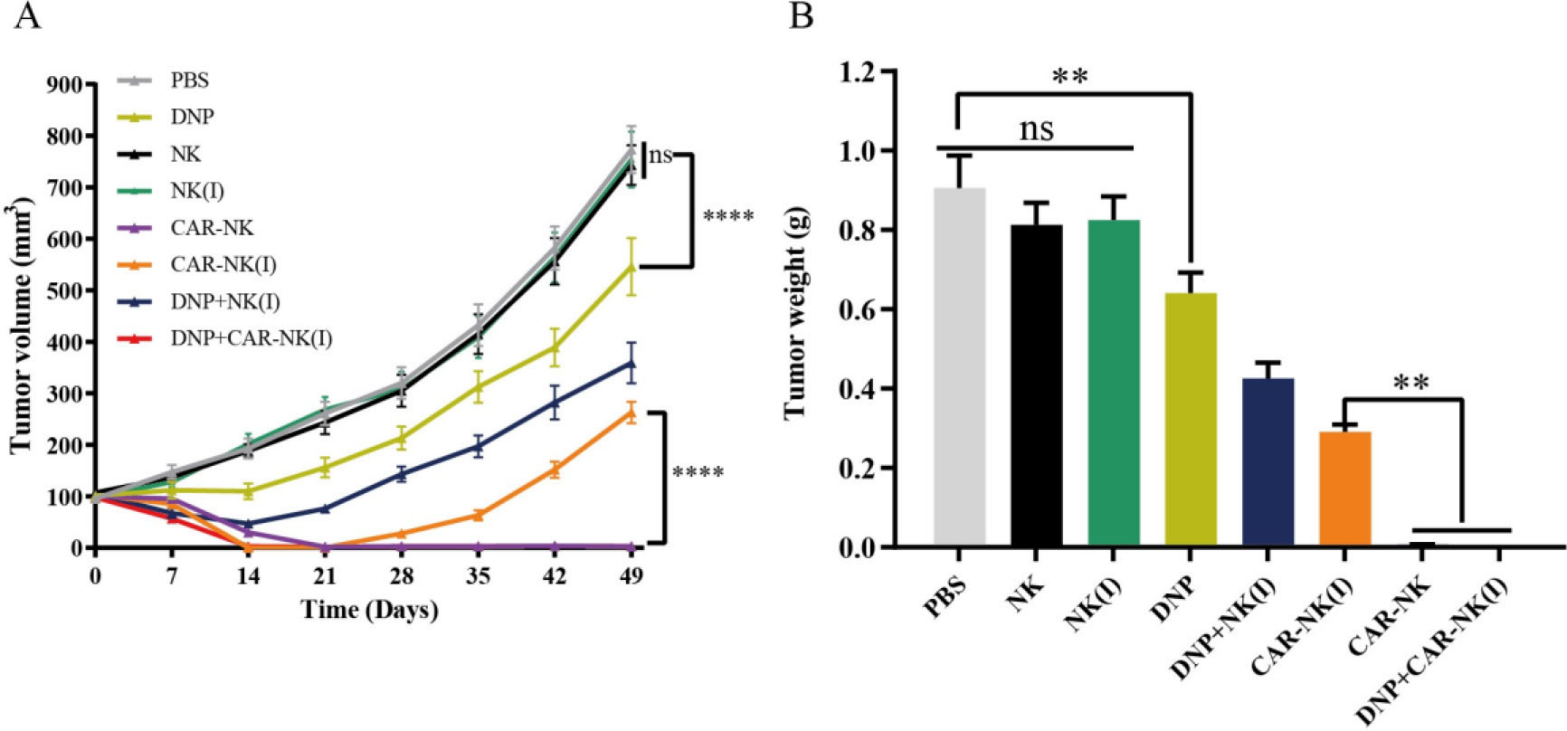
Evaluation of the combined anti-tumor effect of DNP and CAR-NK *in vivo*. **(A)** Tumor growth curves of mice in different treatment groups, tumor volume calculation method V= length× width^2^× 0.5. **(B)** Final tumor weight of different treatment groups. The data were presented as mean ± SEM, and significant differences between groups were measured using one-way analysis of variance (ANOVA). **P <0.01, ****P < 0.0001 ns not significant, n=5.

To evaluate the *in vivo* anti-tumor efficacy of the combined therapeutic strategy, NOD/SCID mice were inoculated with SK-BR-3 cells and treated with various combinations of DNP and Affi1 CAR-NK cells (irradiated and non-irradiated). As shown in **Figure 7A**, non-irradiated Affi1 CAR-NK treatment led to complete tumor regression by day 21. Irradiated CAR-NK cells initially suppressed tumors, but relapse occurred after treatment cessation, highlighting reduced persistence. DNP alone induced transient tumor control, but the combination of DNP and irradiated CAR-NK cells resulted in sustained tumor regression, with some groups achieving complete disappearance (**Figure 7B**). These data suggest that DNP augments the impaired function of irradiated CAR-NK cells, restoring antitumor efficacy.

### 3.8 Biosafety evaluation

**Figure 8:**
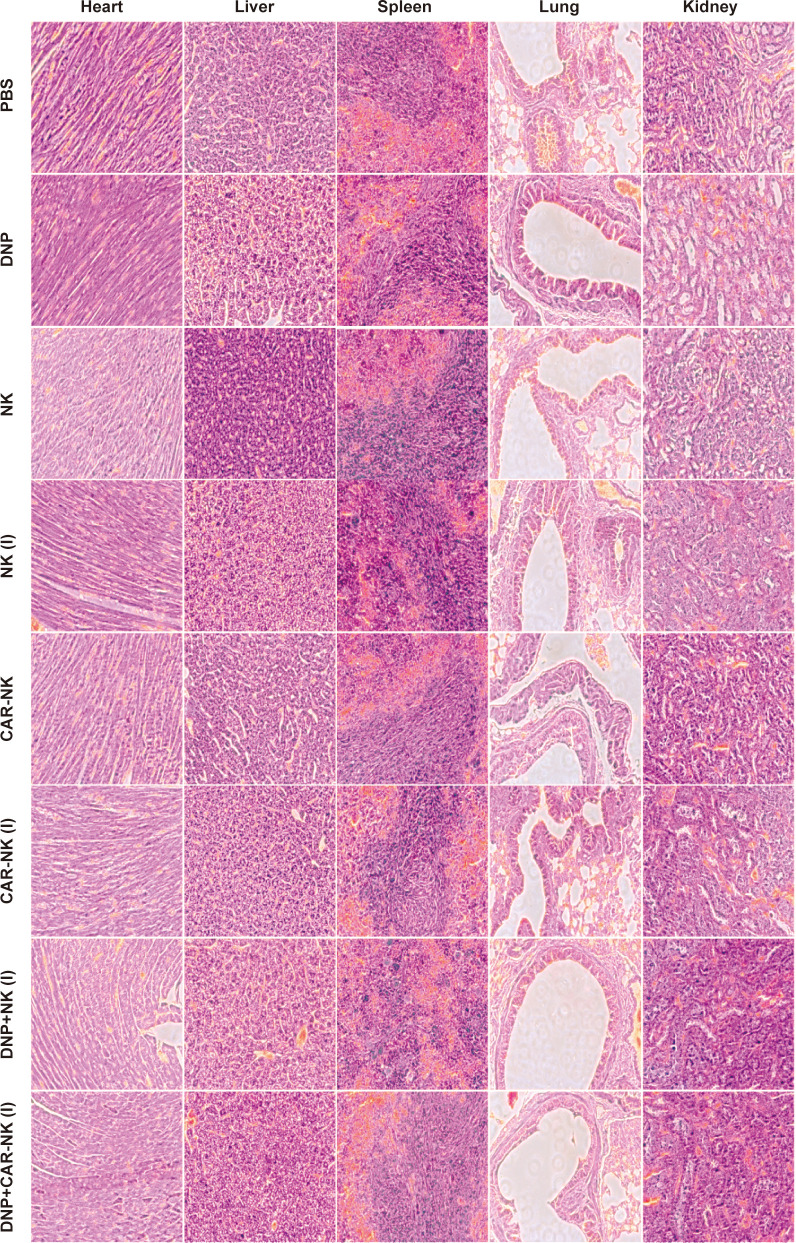
Staining of important organ tissues in mice of different treatment groups. Organ tissues include the heart, liver, spleen, lungs, and kidneys.

The safety of the treatment strategy is crucial for its clinical application. Therefore, we performed a series of biosafety experiments to evaluate the potential toxicity of the combination therapy. During the *in vivo* experiment, we monitored the weight changes of the mice (**Supplementary Figure S4**). The results indicated that none of the treatment methods had a significant impact on the mice’s growth, suggesting that there were no noticeable toxic or side effects associated with the different therapies. At the end of the experiment, we further assessed the presence of residual NK-92MI cells in the peripheral blood of the mice using flow cytometry (**Supplementary Figure S5**). Only a small number of adoptive NK cells were detected in both the NK and CAR-NK groups. In contrast, the percentage of CD45+CD56+ cells in the irradiated groups was comparable to that of the negative control, confirming that no residual NK-92MI cells remained after treatment. Additionally, we conducted histopathological analysis using hematoxylin and eosin (HE) staining on key organs from the mice. As shown in **Figure 8**, the cellular structure and morphology of organs in all treatment groups appeared intact, indicating that both DNP and Affi1 CAR-NK cells did not cause any significant toxicity to normal tissues and organs.

## 4 Discussion

With the development of adoptive immunotherapy, CAR-T therapy has become an effective treatment for cancer. As an innate cytotoxic lymphocyte, NK cells can also be modified with chimeric antigen receptors. Moreover, CAR-NK therapy offers several advantages over CAR-T therapy, including a lower risk of cytokine release syndrome, absence of graft-versus-host disease (GvHD), and potential for “off-the-shelf” application (32). Multiple studies have shown that CAR-NK cells can target a variety of tumor antigens and have achieved certain therapeutic effects in the clinic (33). In addition to donor-derived NK cells, NK92, an established NK cell line, has also shown safety and effectiveness in allogeneic cell therapy and has been successfully used in clinical research (34, 35). Recently, numerous studies have been conducted on the construction of the NK92 cell line as a targeted CAR-NK (36–38). These findings support the feasibility and clinical relevance of cell line–based immunotherapeutic strategies.

Currently, most HER2-targeted CARs have adopted scFvs as their extracellular antigen recognition domain. Such scFv-based CAR-NK cells have been shown to eliminate HER2-positive breast cancer (37, 39) and gliomas (40). At present, the extracellular structure of CAR-NK is mainly scFv, and whether there are other more stable affinity structures needs to be further explored. Here, we successfully constructed the CAR NK92-MI cell line with HER-2 affibody as the extracellular structure, and compared the extracellular region of the affibody with the traditional scFv extracellular domain. The results show that the constructed CAR-NK can successfully express the affibodies, and CAR-NK with affibodies as extracellular recognition domain have similar function to traditional CAR-NK in killing HER-2 positive breast cancer (**Figure 1**). Interestingly, although prior studies have suggested that CAR affinity and epitope location can influence therapeutic efficacy (25), our comparison of three CAR-NK variants-scFv-based and two affibody-based-revealed no clear correlation between affinity magnitude and cytotoxicity. The scFv used here targets domain I of HER2, while the affibodies recognize the junction between domains III and IV. Notably, Affi1 showed stronger cytotoxicity than Affi2, despite recognizing the same epitope. This may be attributed to structural optimizations in Affi1 that enhance thermal stability and hydrophilicity.

Moreover, it is important to note that affibodies, unlike conventional scFvs, exhibit distinct folding patterns and surface charge distributions that may influence receptor accessibility and immune synapse formation. Although Affi1 and Affi2 bind to the same HER2 region, subtle differences in their binding orientation or epitope engagement could result in more favorable spatial positioning of the CAR complex for downstream CD28 and CD3ζ activation in the Affi1 construct. Additionally, enhanced protein stability of Affi1 may promote higher surface expression levels and reduce receptor internalization, thereby improving sustained activation. These findings indicate that factors beyond antigen affinity—such as epitope accessibility, stability, and biophysical conformation—play a critical role in modulating CAR-NK functional activity.

To confirm the functional efficacy of affibody-CARs, real-time live-cell imaging of Affi1-CAR-NK cells co-cultured with SK-BR-3 cells demonstrated robust, targeted cytotoxicity. Collectively, our data provide the first experimental evidence that HER2 affibodies can serve as effective targeting domains in CAR-NK cells, and that their optimization (e.g., via structure-based design) can enhance therapeutic performance.

Considering the biological safety of the NK92-MI cell line, we conducted an exploratory study on the appropriate radiation dose before performing *in vivo* experiments. We find that only CAR-NK after 10 Gy irradiation can completely die after three days, so we chose 10 Gy as the radiation dose before *in vivo* experiments. This is consistent with the safe dose obtained by previous clinical studies on the safety of NK92 cells (35, 40, 41). By observing the effect of irradiation on the secretion of cytokines, we observed that the amount of cytokines secreted by the cells after irradiation decreased accordingly, and the reduction of cytokines decreased the anti-tumor cytotoxicity of CAR-NK. To address this limitation, we investigated the use of chemotherapy for combination therapy. Liposomal doxorubicin formulations have been widely employed in HER2-positive breast cancer and shown to reduce systemic toxicity, particularly cardiotoxicity (42, 43). In our prior work, we developed a doxorubicin nanoparticle (nano-DOX) formulation with improved safety and therapeutic index (29). Based on this foundation, we combined nano-DOX with Affi1-CAR-NK cells for HER2-positive breast cancer therapy and observed a synergistic killing effect against HER2-positive tumor cells. This combination strategy effectively compensated for the irradiated CAR-NK92MI cells’ reduced cytotoxicity without introducing systemic toxicity in internal safety assays. We hypothesize that the synergistic activity arises from enhanced tumor-cell susceptibility following DNP exposure, likely driven by apoptosis priming and TRAIL-receptor pathway activation. This sensitization compensates for the irradiation-induced decline in CAR-NK immune function, resulting in restored and amplified antitumor efficacy.

An essential aspect in advancing NK92-derived CAR-NK cells toward clinical application is achieving an optimal balance between safety assurance and preservation of functional activity after irradiation. Irradiation is required to prevent uncontrolled proliferation and eliminate the risk of tumorigenicity, providing an essential safety safeguard for *in vivo* applications. However, our data demonstrate that irradiation also leads to a dose-dependent decline in cytokine secretion and cytotoxic activity. This indicates that while irradiation effectively induces proliferation arrest, it also compromises the effector functions that are critical for antitumor efficacy. Previous clinical studies have established that 10 Gy irradiation is a widely accepted dose that abrogates proliferation while preserving short-term cytotoxic capacity (13). Consistent with these findings, our results identified 10 Gy as the optimal irradiation dose, as it completely inhibited proliferation within three days. Importantly, we further demonstrated that the combination of nano-doxorubicin with irradiated Affi1-CAR-NK cells restored and even enhanced functional potency, providing a synergistic effect that compensates for irradiation-induced functional decline without compromising safety. These findings highlight that the trade-off between safety and efficacy can be strategically addressed through rational combination therapy.

In conclusion, we successfully constructed the CAR-NK cells using the HER2 affibody as the extracellular receptor and compared the anti-breast cancer effects of the affibody-directed CAR-NK with the traditional scFv-directed CAR-NK. In addition, we found that the combined therapeutic approach of affibody-directed CAR-NK with doxorubicin-loaded nanodrug achieved synergistic treatment effects against HER2-positive breast cancer, indicating an increased anti-tumor effect of targeted chemotherapy for affibody-directed CAR-NK immunotherapy. The present studies thus showed a great potential and a novel approach for HER2-positive breast cancer therapy by using HER2 affibody-directed CAR-NK immunotherapy combined with nano-chemotherapy.

## Data Availability

The raw data supporting the conclusions of this article will be made available by the authors, without undue reservation.

## References

[1] Liu Q Li J Zheng H Yang S Hua Y Huang N . Adoptive cellular immunotherapy for solid neoplasms beyond CAR-T. Mol Cancer. (2023) 22:28. doi: 10.1186/s12943-023-01735-9, PMID: 36750830 PMC9903509

[2] Pu J Liu T Sharma A Jiang L Wei F Ren X . Advances in adoptive cellular immunotherapy and therapeutic breakthroughs in multiple myeloma. Exp Hematol Oncol. (2024) 13:105. doi: 10.1186/s40164-024-00576-6, PMID: 39468695 PMC11514856

[3] Ying Z Huang XF Xiang X Liu Y Kang X Song Y . A safe and potent anti-CD19 CAR T cell therapy. Nat Med. (2019) 25:947–53. doi: 10.1038/s41591-019-0421-7, PMID: 31011207 PMC7518381

[4] Brudno JN Lam N Vanasse D Shen YW Rose JJ Rossi J . Safety and feasibility of anti-CD19 CAR T cells with fully human binding domains in patients with B-cell lymphoma. Nat Med. (2020) 26:270–80. doi: 10.1038/s41591-019-0737-3, PMID: 31959992 PMC7781235

[5] Yip A Webster RM . The market for chimeric antigen receptor T cell therapies. Nat Rev Drug Discov. (2018) 17:161–2. doi: 10.1038/nrd.2017.266, PMID: 29375140

[6] Newick K O'Brien S Moon E Albelda SM . CAR T cell therapy for solid tumors. Annu Rev Med. (2017) 68:139–52. doi: 10.1146/annurev-med-062315-120245, PMID: 27860544

[7] Uslu U June CH . Beyond the blood: expanding CAR T cell therapy to solid tumors. Nat Biotechnol. (2025) 43:506–15. doi: 10.1038/s41587-024-02446-2, PMID: 39533105

[8] Cai Q Warren S Pietrobon V Maeurer M Qi LS Lu TK . Building smart CAR T cell therapies: The path to overcome current challenges. Cancer Cell. (2023) 41:1689–95. doi: 10.1016/j.ccell.2023.08.011, PMID: 37714150

[9] Cheng M C Chen Y Xiao W Sun R Tian Z . NK cell-based immunotherapy for Malignant diseases. Cell Mol Immunol. (2013) 10:230–52. doi: 10.1038/cmi.2013.10, PMID: 23604045 PMC4076738

[10] Fang F Xie S Chen M Li Y Yue J Ma J . Advances in NK cell production. Cell Mol Immunol. (2022) 19:460–81. doi: 10.1038/s41423-021-00808-3, PMID: 34983953 PMC8975878

[11] Ruggeri L Capanni M Urbani E Perruccio K Shlomchik WD Tosti A . Effectiveness of donor natural killer cell alloreactivity in mismatched hematopoietic transplants. Science. (2002) 295:2097–100. doi: 10.1126/science.1068440, PMID: 11896281

[12] Berrien-Elliott MM Jacobs MT Fehniger TA . Allogeneic natural killer cell therapy. Blood. (2023) 141:856–68. doi: 10.1182/blood.2022016200, PMID: 36416736 PMC10023727

[13] Tonn T Schwabe D Klingemann HG Becker S Esser R Koehl U . Treatment of patients with advanced cancer with the natural killer cell line NK-92. CYTOTHERAPY. (2013) 15:1563–70. doi: 10.1016/j.jcyt.2013.06.017, PMID: 24094496

[14] Fabian KP Hodge JW . The emerging role of off-the-shelf engineered natural killer cells in targeted cancer immunotherapy. Mol Ther Oncolytics. (2021) 23:266–76. doi: 10.1016/j.omto.2021.10.001, PMID: 34761106 PMC8560822

[15] Wang W Jiang J Wu C . CAR-NK for tumor immunotherapy: Clinical transformation and future prospects. Cancer Lett. (2020) 472:175–80. doi: 10.1016/j.canlet.2019.11.033, PMID: 31790761

[16] Tam YK Maki G Miyagawa B Hennemann B Tonn T Klingemann HG . Characterization of genetically altered, interleukin 2-independent natural killer cell lines suitable for adoptive cellular immunotherapy. Hum Gene Ther. (1999) 10:1359–73. doi: 10.1089/10430349950018030, PMID: 10365666

[17] Jayaraman J Mellody MP Hou AJ Desai RP Fung AW Pham AHT . CAR-T design: Elements and their synergistic function. EBioMedicine. (2020) 58:102931. doi: 10.1016/j.ebiom.2020.102931, PMID: 32739874 PMC7393540

[18] Mazinani M Rahbarizadeh F . CAR-T cell potency: from structural elements to vector backbone components. biomark Res. (2022) 10:70. doi: 10.1186/s40364-022-00417-w, PMID: 36123710 PMC9487061

[19] Nix MA Wiita AP . Alternative target recognition elements for chimeric antigen receptor (CAR) T cells: beyond standard antibody fragments. Cytotherapy. (2024) 26:729–38. doi: 10.1016/j.jcyt.2024.02.024, PMID: 38466264

[20] Han X Cinay GE Zhao Y Guo Y Zhang X Wang P . Adnectin-based design of chimeric antigen receptor for T cell engineering. Mol Ther. (2017) 25:2466–76. doi: 10.1016/j.ymthe.2017.07.009, PMID: 28784559 PMC5675441

[21] Patasic L Seifried J Bezler V Kaljanac M Schneider IC Schmitz H . Designed Ankyrin Repeat Protein (DARPin) to target chimeric antigen receptor (CAR)-redirected T cells towards CD4+ T cells to reduce the latent HIV+ cell reservoir. Med Microbiol Immunol. (2020) 209:681–91. doi: 10.1007/s00430-020-00692-0, PMID: 32918599 PMC7568711

[22] Ståhl S Gräslund T Eriksson Karlström A Frejd FY Nygren PÅ Löfblom J . Affibody molecules in biotechnological and medical applications. Trends Biotechnol. (2017) 35:691–712. doi: 10.1016/j.tibtech.2017.04.007, PMID: 28514998

[23] Morales-Kastresana A Siegemund M Haak S Peper-Gabriel J Neiens V Rothe C . Anticalin®-based therapeutics: Expanding new frontiers in drug development. Int Rev Cell Mol Biol. (2022) 369:89–106. doi: 10.1016/bs.ircmb.2022.03.009, PMID: 35777866

[24] Mo F Duan S Jiang X Yang X Hou X Shi W . Nanobody-based chimeric antigen receptor T cells designed by CRISPR/Cas9 technology for solid tumor immunotherapy. Signal Transduct Target Ther. (2021) 6:80. doi: 10.1038/s41392-021-00462-1, PMID: 33627635 PMC7904846

[25] Baum RP Prasad V Müller D Schuchardt C Orlova A Wennborg A . Molecular imaging of HER2-expressing Malignant tumors in breast cancer patients using synthetic 111In- or 68Ga-labeled affibody molecules. J Nucl Med. (2010) 51:892–7. doi: 10.2967/jnumed.109.073239, PMID: 20484419

[26] Xia X Gao W Yang X Huang W Xia XX Yan D . Chemically engineered affinity protein drugs for covalent targeted cancer therapy. J Am Chem Soc. (2025) 147:19687–701. doi: 10.1021/jacs.5c02212, PMID: 40445865

[27] Feldwisch J Tolmachev V Lendel C Herne N Sjöberg A Larsson B . Design of an optimized scaffold for affibody molecules. J Mol Biol. (2010) 398:232–47. doi: 10.1016/j.jmb.2010.03.002, PMID: 20226194

[28] Shafei A El-Bakly W Sobhy A Wagdy O Reda A Aboelenin O . A review on the efficacy and toxicity of different doxorubicin nanoparticles for targeted therapy in metastatic breast cancer. BioMed Pharmacother. (2017) 95:1209–18. doi: 10.1016/j.biopha.2017.09.059, PMID: 28931213

[29] Deng X Cao M Zhang J Hu K Yin Z Zhou Z . Hyaluronic acid-chitosan nanoparticles for co-delivery of MiR-34a and doxorubicin in therapy against triple negative breast cancer. BIOMATERIALS. (2014) 35:4333–44. doi: 10.1016/j.biomaterials.2014.02.006, PMID: 24565525

[30] Cheng LS Liu AP Yang JH Dong YQ Li LW Wang J . Construction, expression and characterization of the engineered antibody against tumor surface antigen, p185(c-erbB-2). Cell Res. (2003) 13:35–48. doi: 10.1038/sj.cr.7290149, PMID: 12643348

[31] Eigenbrot C Ultsch M Dubnovitsky A Abrahmsen L Hard T . Structural basis for high-affinity HER2 receptor binding by an engineered protein. Proc Natl Acad Sci U.S.A. (2010) 107:15039–44. doi: 10.1073/pnas.1005025107, PMID: 20696930 PMC2930565

[32] Wennerberg E Sarhan D Carlsten M Kaminskyy VO D'Arcy P Zhivotovsky B . Doxorubicin sensitizes human tumor cells to NK cell- and T-cell-mediated killing by augmented TRAIL receptor signaling. Int J Cancer. (2013) 133:1643–52. doi: 10.1002/ijc.28163, PMID: 23504627 PMC8725935

[33] Rana S Thomas L Kirkham AM Shorr R Visram A Maganti H . CAR-NK cells to treat patients with cancer: a systematic scoping review of published studies and registered clinical trials. Cytotherapy. (2025) 27:1179–89. doi: 10.1016/j.jcyt.2025.06.006, PMID: 40632045

[34] Klingemann H . The NK-92 cell line-30 years later: its impact on natural killer cell research and treatment of cancer. Cytotherapy. (2023) 25:451–7. doi: 10.1016/j.jcyt.2022.12.003, PMID: 36610812

[35] Arai S Meagher R Swearingen M Myint H Rich E Martinson J . Infusion of the allogeneic cell line NK-92 in patients with advanced renal cell cancer or melanoma: a phase I trial. CYTOTHERAPY. (2008) 10:625–32. doi: 10.1080/14653240802301872, PMID: 18836917

[36] Peng Y Zhang W Chen Y Zhang L Shen H Wang Z . Engineering c-Met-CAR NK-92 cells as a promising therapeutic candidate for lung adenocarcinoma. Pharmacol Res. (2023) 188:106656. doi: 10.1016/j.phrs.2023.106656, PMID: 36640859

[37] Romanski A Uherek C Bug G Seifried E Klingemann H Wels WS . CD19-CAR engineered NK-92 cells are sufficient to overcome NK cell resistance in B-cell Malignancies. J Cell Mol Med. (2016) 20:1287–94. doi: 10.1111/jcmm.12810, PMID: 27008316 PMC4929308

[38] Biggi AFB Silvestre RN Tirapelle MC de Azevedo JTC García HDM Henrique Dos Santos M . IL-27-engineered CAR.19-NK-92 cells exhibit enhanced therapeutic efficacy. Cytotherapy. (2024) 26:1320–30. doi: 10.1016/j.jcyt.2024.06.001, PMID: 38970613

[39] Oelsner S Friede ME Zhang C Wagner J Badura S Bader P . Continuously expanding CAR NK-92 cells display selective cytotoxicity against B-cell leukemia and lymphoma. CYTOTHERAPY. (2017) 19:235–49. doi: 10.1016/j.jcyt.2016.10.009, PMID: 27887866

[40] Zhang C Burger MC Jennewein L Genßler S Schönfeld K Zeiner P . ErbB2/HER2-specific NK cells for targeted therapy of glioblastoma. J Natl Cancer Inst. (2016) 108. doi: 10.1093/jnci/djv375, PMID: 26640245

[41] Zhang C Hu Y Shi C . Targeting natural killer cells for tumor immunotherapy. Front Immunol. (2020) 11:60. doi: 10.3389/fimmu.2020.00060, PMID: 32140153 PMC7042203

[42] Chia S Clemons M Martin LA Rodgers A Gelmon K Pond GR . Pegylated liposomal doxorubicin and trastuzumab in HER-2 overexpressing metastatic breast cancer: a multicenter phase II trial. J Clin Oncol. (2006) 24:2773–8. doi: 10.1200/JCO.2005.03.8331, PMID: 16682726

[43] Cortes J Di Cosimo S Climent MA Cortés-Funes H Lluch A Gascón P . Nonpegylated liposomal doxorubicin (TLC-D99), paclitaxel, and trastuzumab in HER-2-overexpressing breast cancer: a multicenter phase I/II study. Clin Cancer Res. (2009) 15:307–14. doi: 10.1158/1078-0432.CCR-08-1113, PMID: 19118059

