# Supplementary material for: Construction of anti-HER2 affibody-directed CAR-NK and its synergistic effects with doxorubicin-loaded nanodrug against HER2-positive breast cancer
Source: Front Immunol. 2026 Jan 12;16:1692107. doi: 10.3389/fimmu.2025.1692107 (PMC12832660; doi:10.3389/fimmu.2025.1692107)
Supplement: Supplementary file 1 [file Presentation1.pptx]

## Slide 1
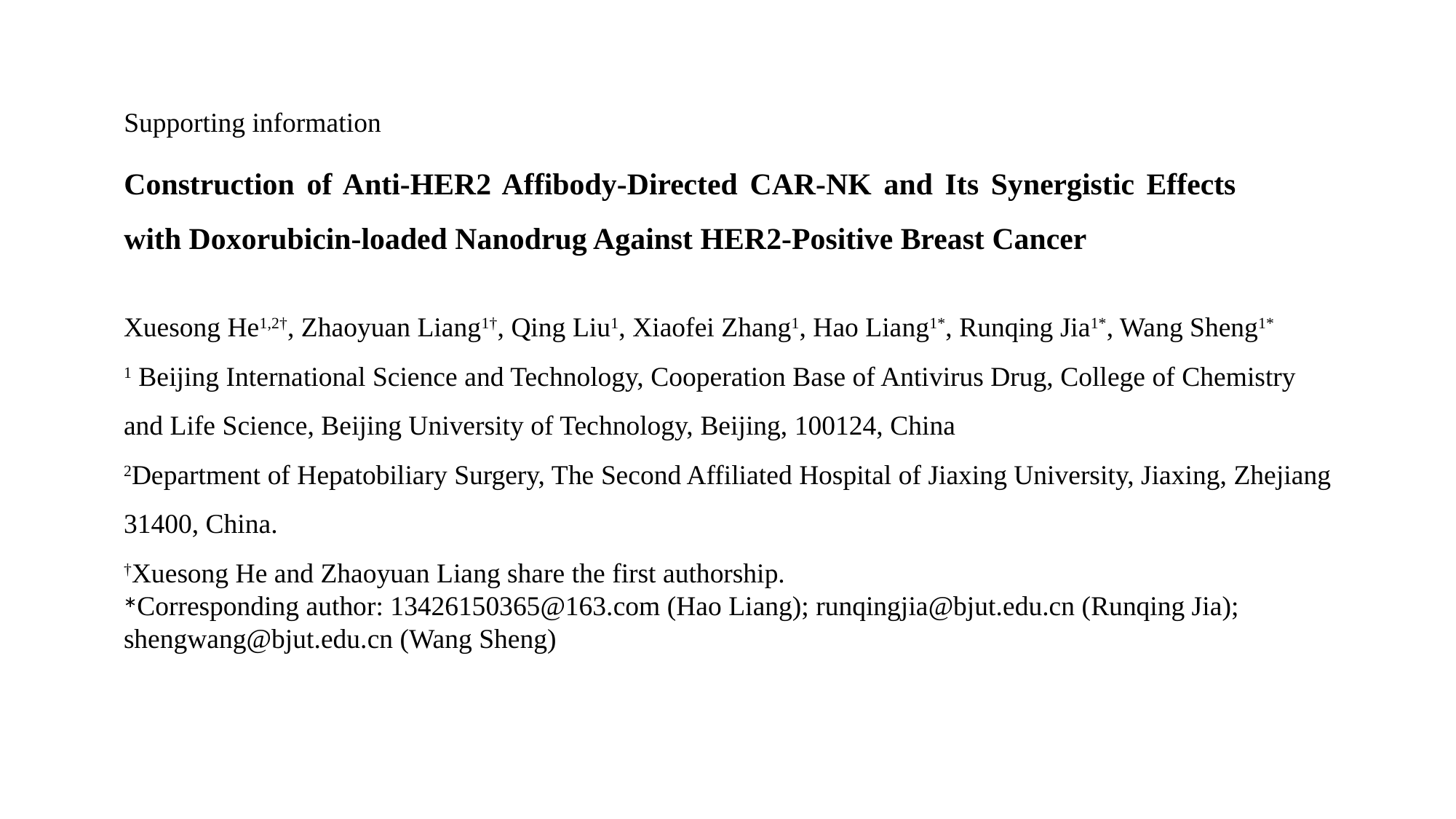

Supporting information
Construction of Anti-HER2 Affibody-Directed CAR-NK and Its Synergistic Effects with Doxorubicin-loaded Nanodrug Against HER2-Positive Breast Cancer
Xuesong He1,2†, Zhaoyuan Liang1†, Qing Liu1, Xiaofei Zhang1, Hao Liang1*, Runqing Jia1*, Wang Sheng1*
1 Beijing International Science and Technology, Cooperation Base of Antivirus Drug, College of Chemistry and Life Science, Beijing University of Technology, Beijing, 100124, China
2Department of Hepatobiliary Surgery, The Second Affiliated Hospital of Jiaxing University, Jiaxing, Zhejiang 31400, China.
†Xuesong He and Zhaoyuan Liang share the first authorship.
∗Corresponding author: 13426150365@163.com (Hao Liang); runqingjia@bjut.edu.cn (Runqing Jia); shengwang@bjut.edu.cn (Wang Sheng)

## Slide 2
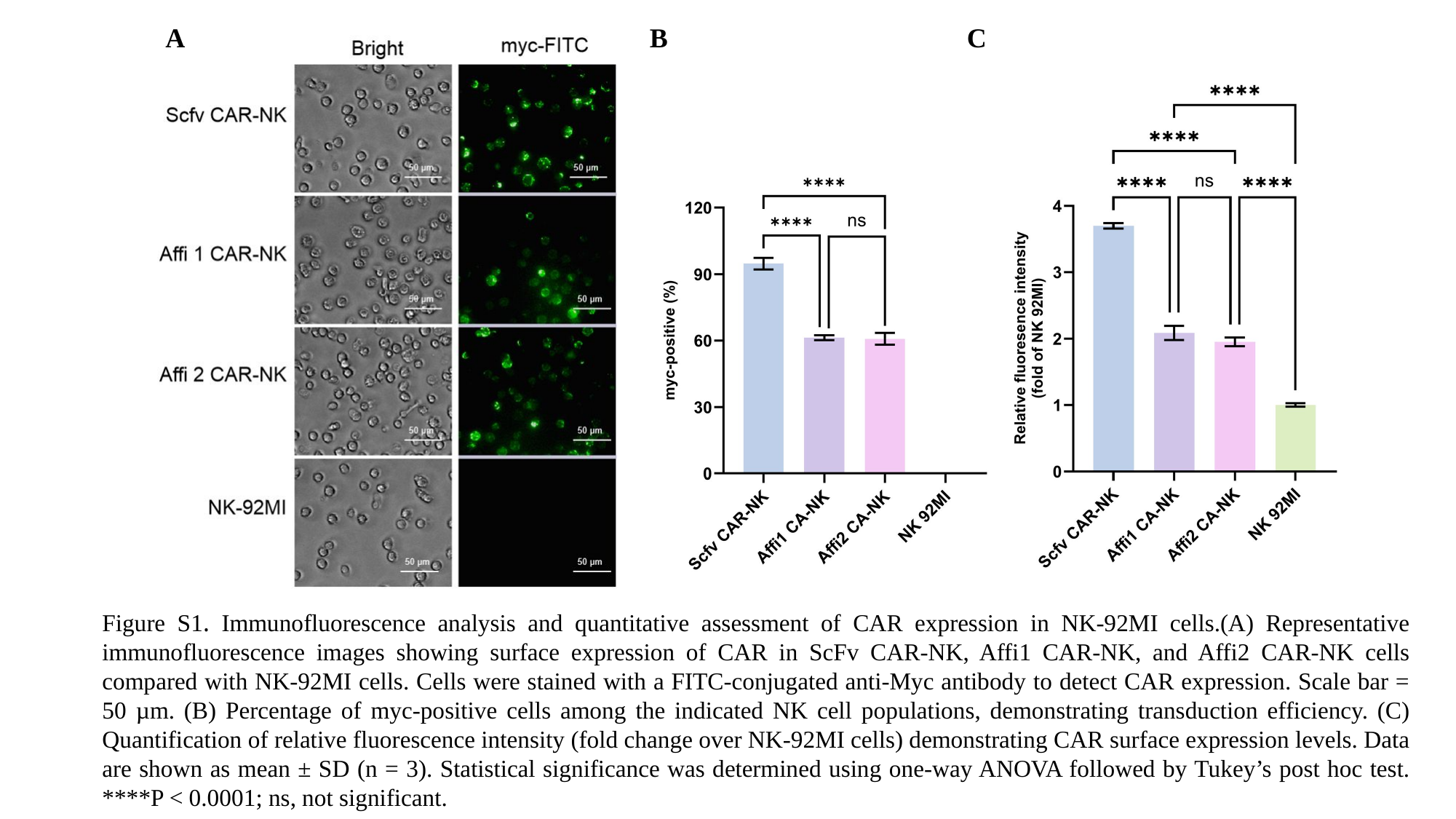

A
B
C
Figure S1. Immunofluorescence analysis and quantitative assessment of CAR expression in NK-92MI cells.(A) Representative immunofluorescence images showing surface expression of CAR in ScFv CAR-NK, Affi1 CAR-NK, and Affi2 CAR-NK cells compared with NK-92MI cells. Cells were stained with a FITC-conjugated anti-Myc antibody to detect CAR expression. Scale bar = 50 µm. (B) Percentage of myc-positive cells among the indicated NK cell populations, demonstrating transduction efficiency. (C) Quantification of relative fluorescence intensity (fold change over NK-92MI cells) demonstrating CAR surface expression levels. Data are shown as mean ± SD (n = 3). Statistical significance was determined using one-way ANOVA followed by Tukey’s post hoc test. ****P < 0.0001; ns, not significant.

## Slide 3
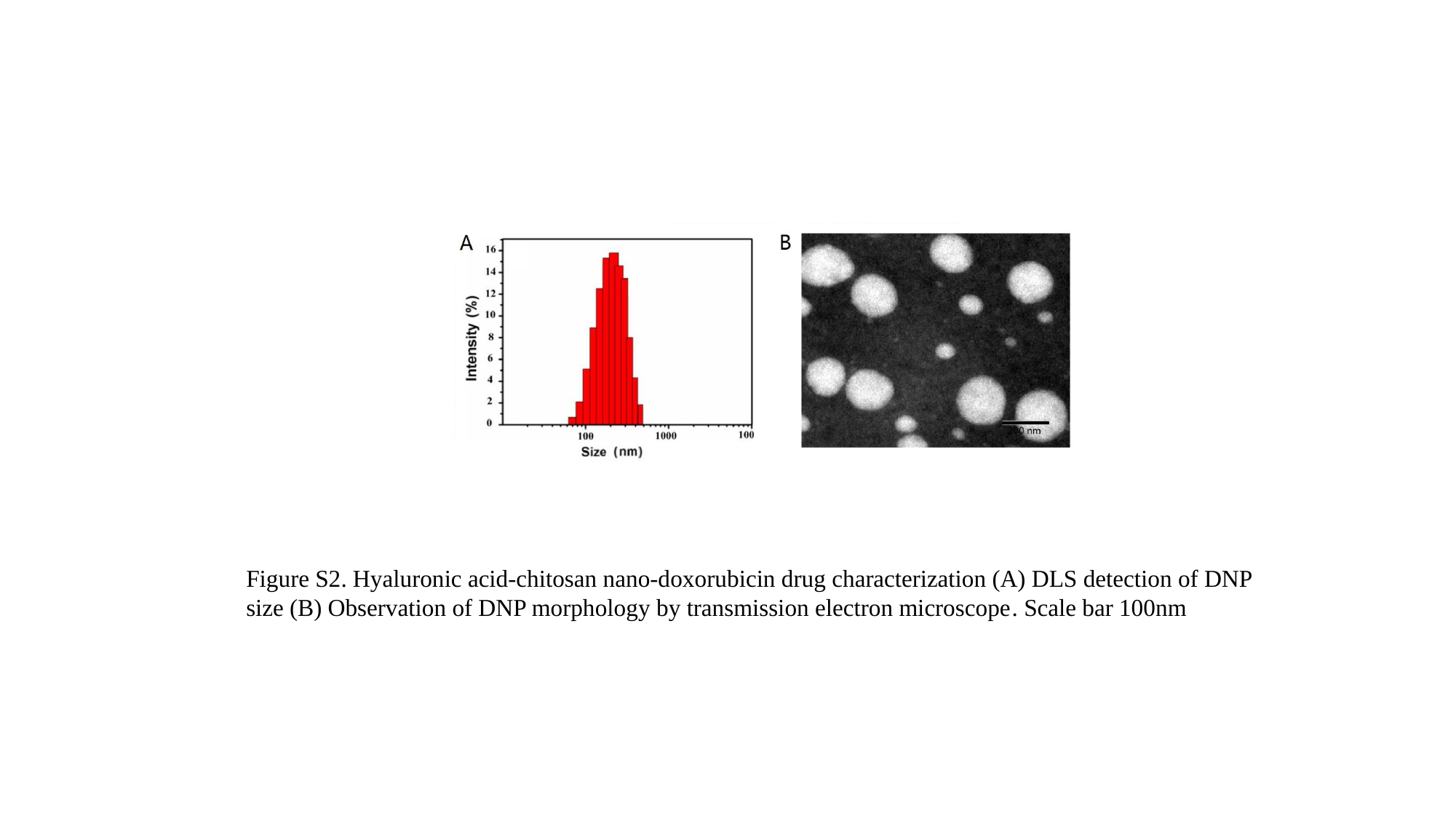

Figure S2. Hyaluronic acid-chitosan nano-doxorubicin drug characterization (A) DLS detection of DNP size (B) Observation of DNP morphology by transmission electron microscope. Scale bar 100nm

## Slide 4
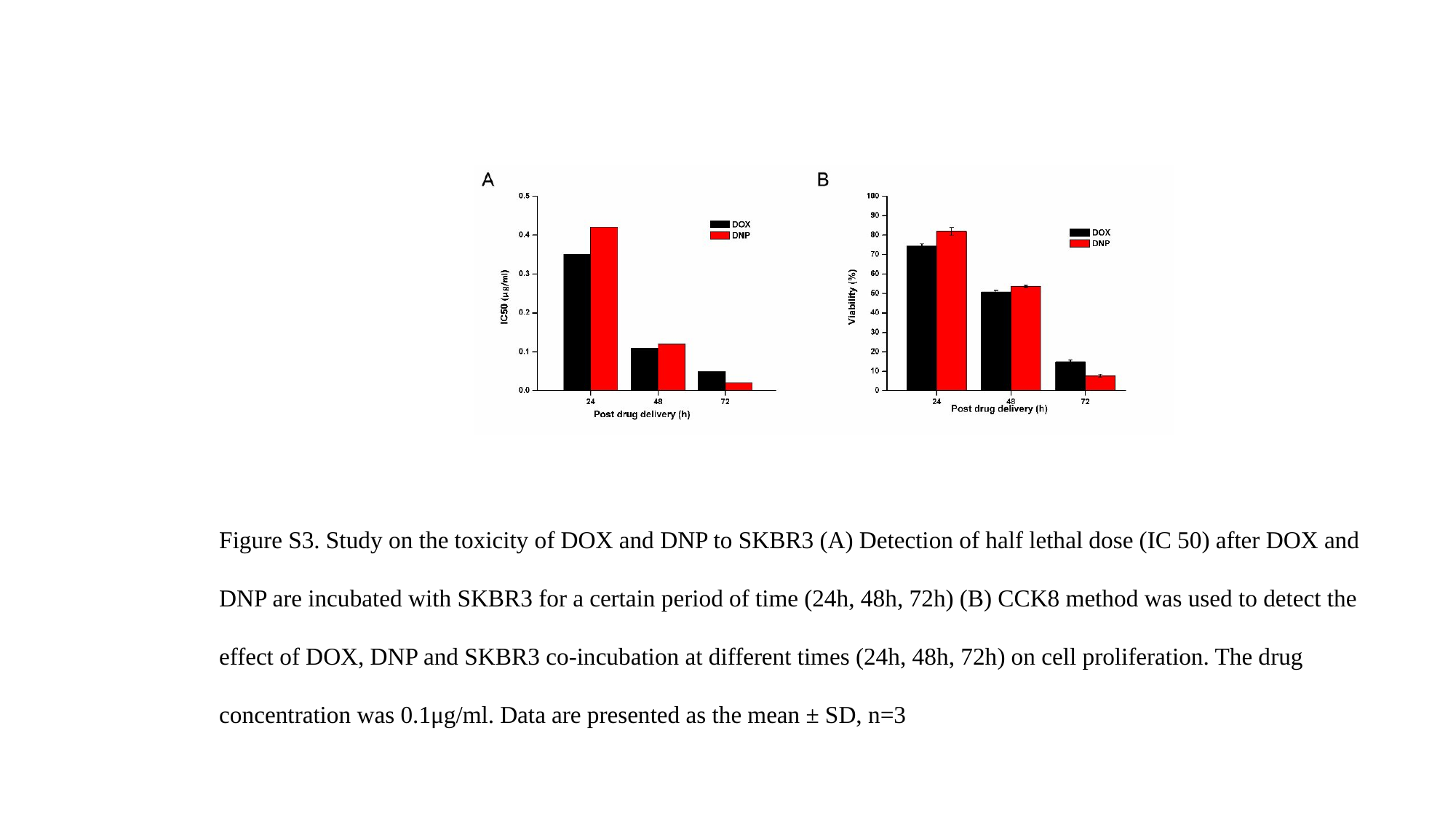

Figure S3. Study on the toxicity of DOX and DNP to SKBR3 (A) Detection of half lethal dose (IC 50) after DOX and DNP are incubated with SKBR3 for a certain period of time (24h, 48h, 72h) (B) CCK8 method was used to detect the effect of DOX, DNP and SKBR3 co-incubation at different times (24h, 48h, 72h) on cell proliferation. The drug concentration was 0.1μg/ml. Data are presented as the mean ± SD, n=3

## Slide 5
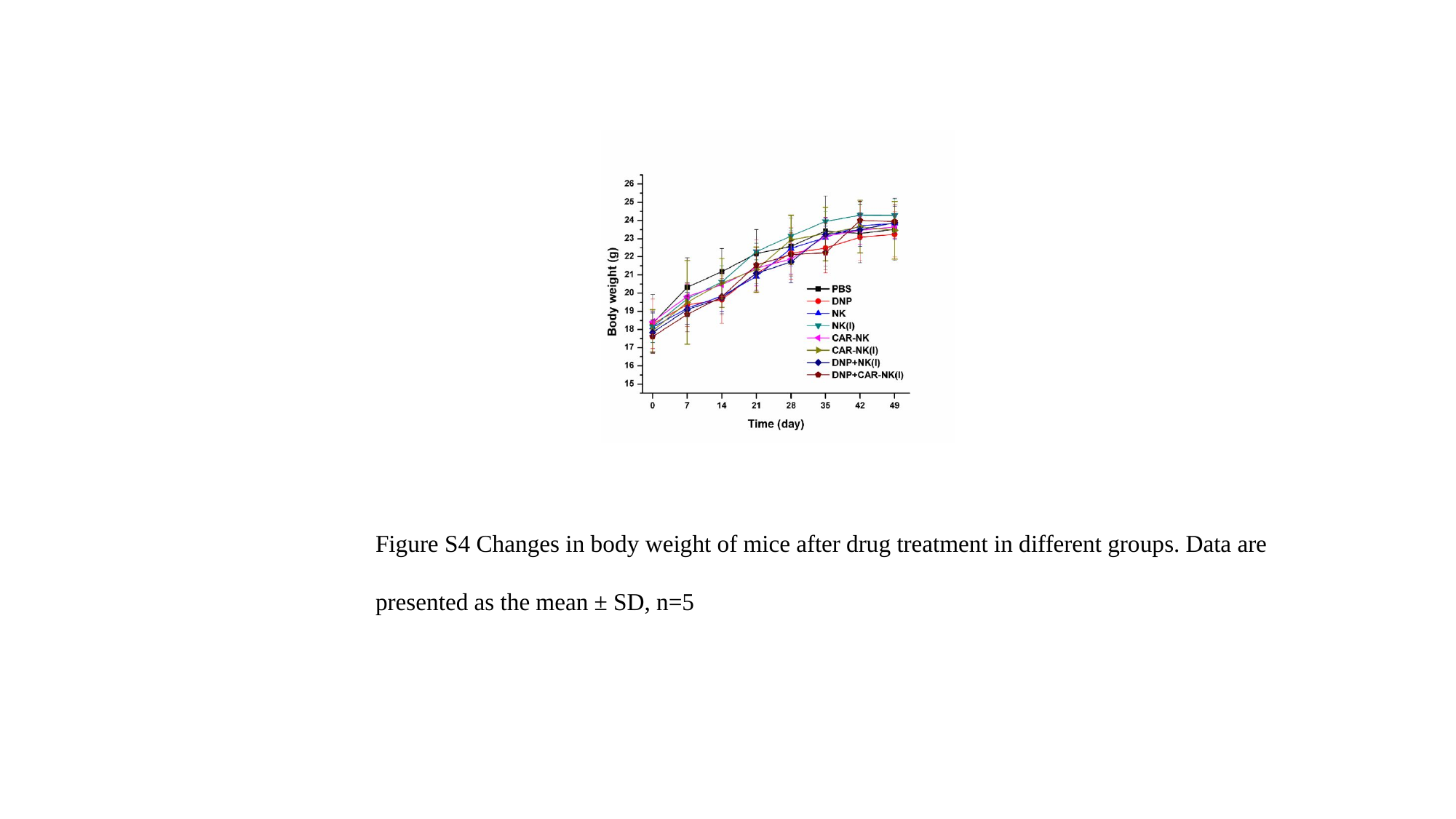

Figure S4 Changes in body weight of mice after drug treatment in different groups. Data are presented as the mean ± SD, n=5

## Slide 6
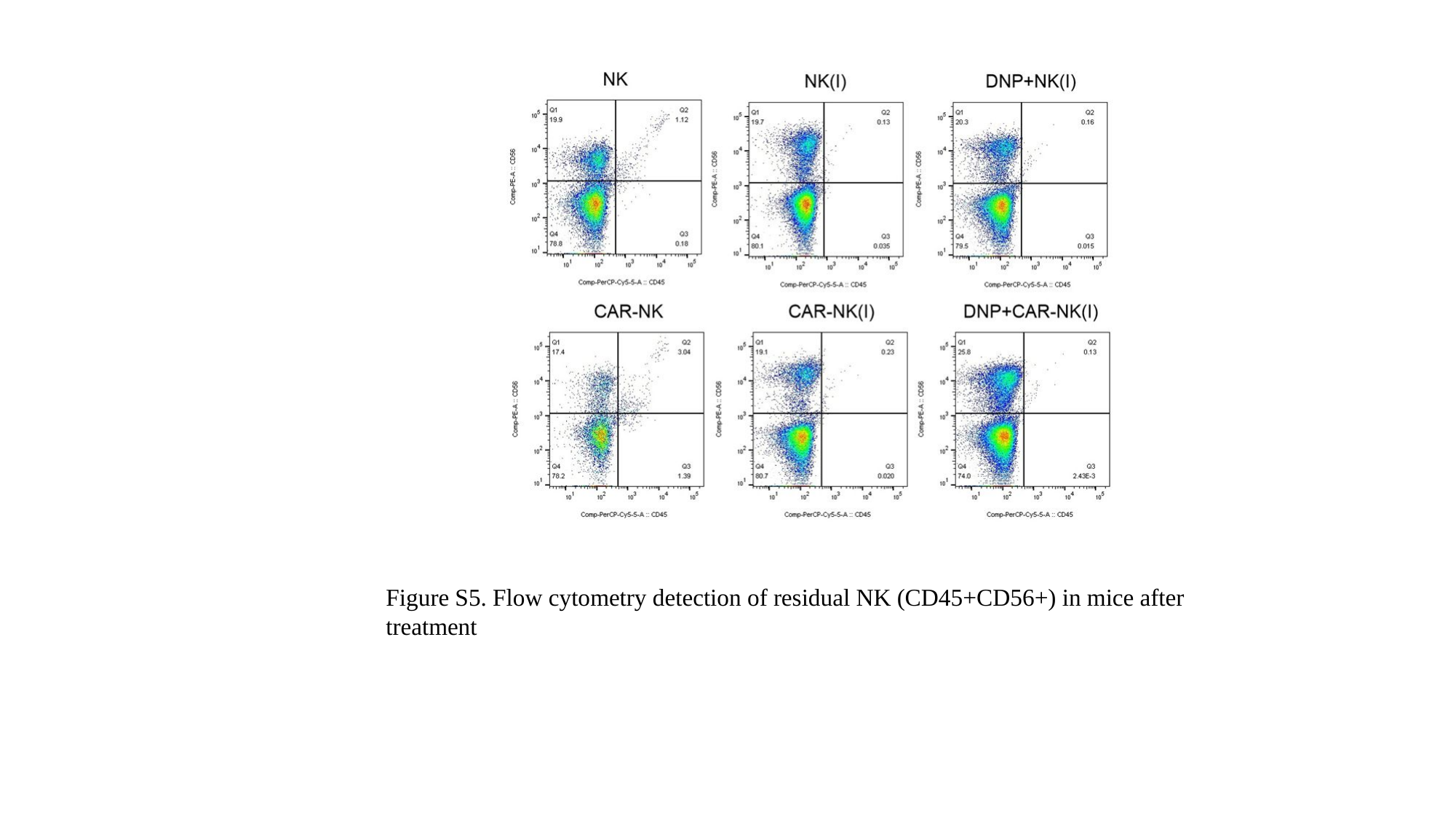

Figure S5. Flow cytometry detection of residual NK (CD45+CD56+) in mice after treatment

## Slide 7
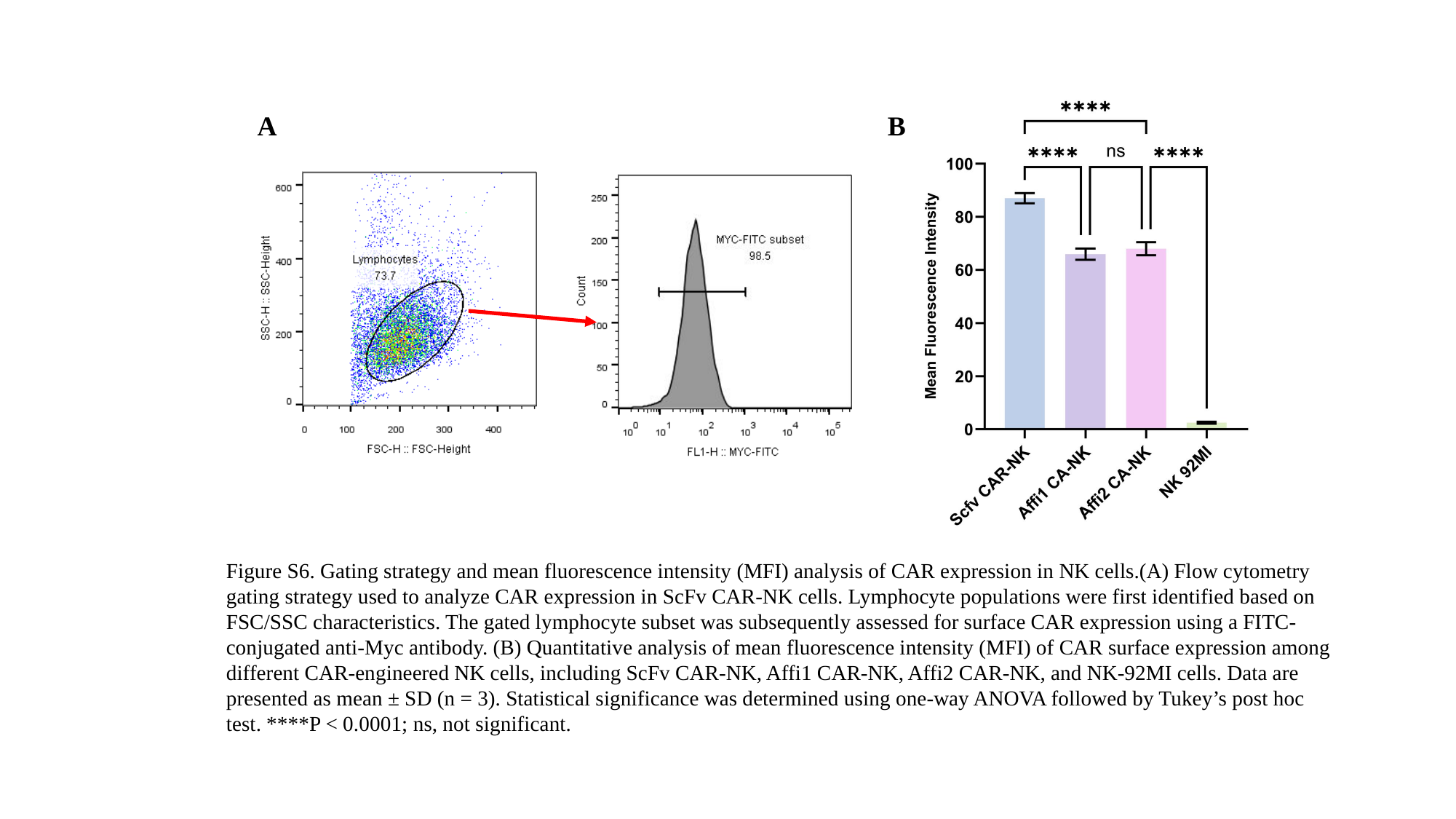

A
B
Figure S6. Gating strategy and mean fluorescence intensity (MFI) analysis of CAR expression in NK cells.(A) Flow cytometry gating strategy used to analyze CAR expression in ScFv CAR-NK cells. Lymphocyte populations were first identified based on FSC/SSC characteristics. The gated lymphocyte subset was subsequently assessed for surface CAR expression using a FITC-conjugated anti-Myc antibody. (B) Quantitative analysis of mean fluorescence intensity (MFI) of CAR surface expression among different CAR-engineered NK cells, including ScFv CAR-NK, Affi1 CAR-NK, Affi2 CAR-NK, and NK-92MI cells. Data are presented as mean ± SD (n = 3). Statistical significance was determined using one-way ANOVA followed by Tukey’s post hoc test. ****P < 0.0001; ns, not significant.

## Slide 8
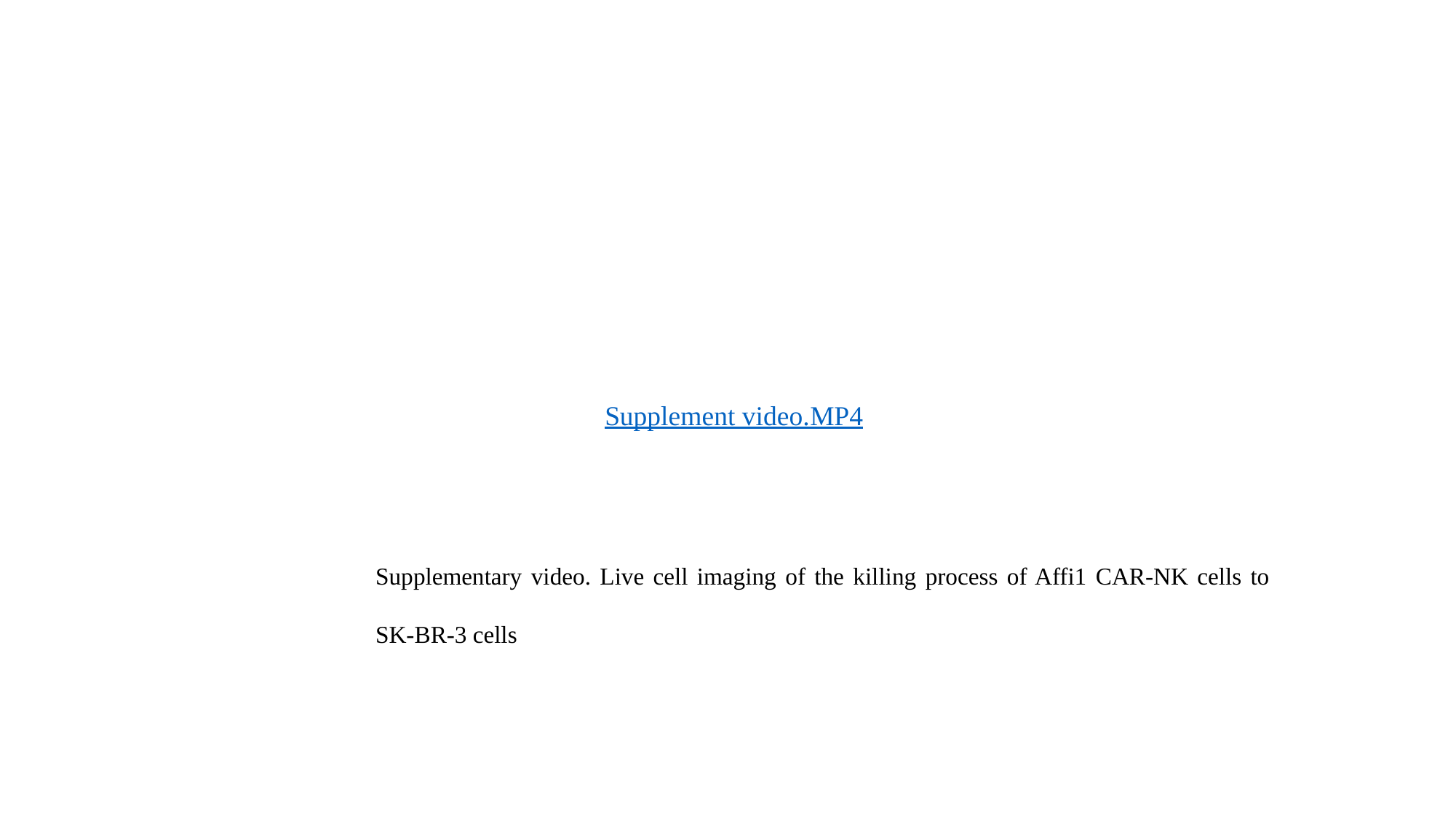

Supplement video.MP4
Supplementary video. Live cell imaging of the killing process of Affi1 CAR-NK cells to SK-BR-3 cells
